# Germline selection of *PTPN11* (HGNC:9644) variants make a major contribution to both Noonan syndrome's high birth rate and the transmission of sporadic cancer variants resulting in fetal abnormality

**DOI:** 10.1002/humu.24493

**Published:** 2022-11-24

**Authors:** Jordan Eboreime, Soo‐Kyung Choi, Song‐Ro Yoon, Anastasiia Sadybekov, Vsevolod Katritch, Peter Calabrese, Norman Arnheim

**Affiliations:** ^1^ Department of Biological Sciences Molecular and Computational Biology Program, University of Southern California Los Angeles California USA; ^2^ Department of Chemistry Bridge Institute, University of Southern California Los Angeles California USA; ^3^ Department of Quantitative and Computational Biology University of Southern California Los Angeles California USA

**Keywords:** fetal abnormality, germline selection, Noonan syndrome, RASopathies, sporadic cancer, SSC clonal expansion

## Abstract

Some spontaneous germline gain‐of‐function mutations promote spermatogonial stem cell clonal expansion and disproportionate variant sperm production leading to unexpectedly high transmission rates for some human genetic conditions. To measure the frequency and spatial distribution of de novo mutations we divided three testes into 192 pieces each and used error‐corrected deep‐sequencing on each piece. We focused on *PTPN11* (HGNC:9644) Exon 3 that contains 30 different *PTPN11* Noonan syndrome (NS) mutation sites. We found 14 of these variants formed clusters among the testes; one testis had 11 different variant clusters. The mutation frequencies of these different clusters were not correlated with their case‐recurrence rates nor were case recurrence rates of *PTPN11* variants correlated with their tyrosine phosphatase levels thereby confusing *PTPN11*'s role in germline clonal expansion. Six of the *PTPN11* exon 3 de novo variants associated with somatic mutation‐induced sporadic cancers (but not NS) also formed testis clusters. Further, three of these six variants were observed among fetuses that underwent prenatal ultrasound screening for NS‐like features. Mathematical modeling showed that germline selection can explain both the mutation clusters and the high incidence of NS (1/1000–1/2500).

## Graphical abstract

Mutation frequency heat map of spontaneous *PTPN11* variants show spatial clustering in the testis due to germline selection explaining the high birth incidence of Noonan syndrome. Some somatic *PTPN11* cancer mutations are similarly clustered in testis.

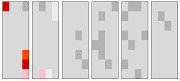

## 1 BACKGROUND

Some genetic disorders arise every generation from de novo germline gain‐of‐function variants. For some of these conditions, the incidence of spontaneous cases would require a germline base substitution rate 100–1000 times greater than the genome average (~10^−8^, reviewed in Nachman & Crowell, 2000; Rahbari et al., 2016; Ségurel et al., 2014). Considerable evidence from testes (Choi et al., 2008, 2012; Maher et al., 2016a, 2016b, 2018; Qin et al., 2007; Shinde et al., 2013; Yoon et al., 2013) and sperm (Giannoulatou et al., 2013; Goriely et al., 2003, 2005, 2009; Yoon et al., 2009) studies suggests these amino acid changes, although detrimental to the offspring, provide a selective advantage to the newly mutated spermatogonial stem cell (SSC) compared to SSCs without the variant (reviewed in Arnheim & Calabrese, 2009, 2016; Goriely & Wilkie, 2012; Maher et al., 2016b). The resulting disproportionate production of mutant sperm increases their probability of transmission to the next generation. These detrimental variants have been called Paternal age effect (Goriely & Wilkie, 2012) or RAMP variants (Yoon et al., 2013) (RAMP signifies they **r** ecur in different SSC at the same nucleotide site, are inherited in an **a** utosomal dominant fashion, originate almost exclusively in the **m** ale germline, and lead to an increase in sperm variant frequency with **p** aternal age). Examples of genes that acquire such deleterious RAMP/PAE variants are *FGFR2* (*HGNC:3689*), *FGFR3* (*HGNC:3690*), *RET* (*HGNC:9967*), *HRAS* (*HGNC:5173*) and *PTPN11* (*HGNC:9644*) (reviewed in Arnheim & Calabrese, 2009, 2016; Goriely & Wilkie, 2012). These variants promote excessive signaling through the RAS/mitogen‐activated protein kinase (RAS/MAPK) and related pathways (Araki et al., 2003; Castinetti et al., 2018; Dance et al., 2008; Fragale et al., 2004; Gripp & Lin, 2012; Ornitz & Itoh, 2015).

Molecular details on how changes in SSC signaling promote a germline selective advantage are lacking. To provide more insight into these processes we studied a variety of variants that cause Noonan syndrome (NS), a common Mendelian genetic condition occurring once in every 1000–2500 births (Allanson & Roberts, 2001 Nov 15 [Updated February 25, 2016]; Tartaglia et al., 2010; Zenker, 2017). Clinical signs of NS include characteristic facial dysmorphism, short stature, congenital heart disease, intellectual disability/delay, and a slight predisposition to acquire usually transient newborn/infant blood cell cancers. NS is the most frequent among the developmental disorders known as RASopathies (Allanson & Roberts, 2001 Nov 15 [Updated February 25, 2016]; Rauen, 2013). This complex group of conditions exhibit overlapping clinical signs and are caused by variants that lead to dysfunction of the RAS/MAPK signaling pathway.

The NS1 form (MIM# 163950) of NS results from mutations (Maheshwari et al., 2002; Tartaglia et al., 2001, 2002) in *PTPN11* and accounts for 50%–60% of all newborn NS cases (Tartaglia & Gelb, 2005; Zenker, 2017). A significant proportion (>50%) of these occur de novo in the unaffected father's germline (Tartaglia et al., 2001, 2004, 2006); the rest are transmitted by affected family members. The *PTPN11* transcript encodes the Tyrosine phosphatase nonreceptor type 11 protein PTPN11 commonly called SHP‐2. SHP‐2 is present ubiquitously and its role in signaling is critical to animal development and general organismal homeostasis (Araki et al., 2003; Dance et al., 2008; Fragale et al., 2004; Keilhack et al., 2005) including mouse SSC self‐renewal and differentiation (Puri & Walker, 2016; Puri et al., 2014).

The European Network on NS and related disorders database (Zenker, 2017) (NSEuroNet, https://nseuronet.com) lists 110 different annotated NS1 missense variant sites reported in the 593 amino acid coding region of SHP‐2. NSEuroNet reflects an earlier compilation of NS1 variants (Tartaglia et al., 2006) thereby providing a reasonable picture of the relative birth rate variation among different *PTPN11* variants. According to NSEuroNet the number of reported cases at the NS1 variant sites range from 1 to 241.

Previous work on germline selection showed that RAMP/PAE variants form testis clusters in virtually all men beginning in their late 30s. Further, a single testis can harbor multiple mutation clusters each initially derived from a single SSC RAMP/PAE variant that evolves independently in the testis (Arnheim & Calabrese, 2016; Choi et al., 2008, 2012; Maher et al., 2016a, 2018; Qin et al., 2007; Shinde et al., 2013; Yoon et al., 2013). The lack of clusters in young men indicated clonal expansion of mutated SSC primarily occurs in adulthood and not during development (Arnheim & Calabrese, 2016; Choi et al., 2008; Goriely & Wilkie, 2012) consistent with the paternal age effect as inferred from earlier work on sperm (Goriely et al., 2003, 2005).

To further examine the role of germline selection we used error‐corrected, massively parallel, DNA deep‐sequencing (safe sequencing system [SSS] [Eboreime et al., 2016; Kinde et al., 2011]) targeting *PTPN11* exon 3 (hereafter E3). E3 spans amino acids 46–82 in SHP‐2 and contains 108 coding nucleotides including 30 NS1 sites (~27% of all known NS1 missense variant sites) with varying case recurrence rates (Zenker, 2017). We used the E3 data to study the potential for selection‐driven testis cluster formation among the multiple NS1 variant sites. Some E3 *PTPN11* variants are also responsible for sporadic hematopoietic and lymphoid neoplasms (H&LN) in the general population that typically arise only from somatic mutations (see COSMIC v94, Catalogue Of Somatic Variants In *C* ancer, https://cancer.sanger.ac.uk/). We found germline testis clusters carrying sporadic‐only (S‐O) cancer variants (also see Maher et al., 2018). Our results also contribute to a nuanced insight into the germline selection process and the role of germline sporadic cancer NS1 variants in early fetal and possibly embryonic death. Finally, mathematical modeling explained the unusually high birth frequency of NS using our *PTPN11* E3 data.

## 2 METHODS

### 2.1 Reference sequences and databases

The Accession and Version numbers for the *PTPN11* transcipt and PTPN11 protein are (RefSeq NM_002834.5) and (RefSeq NP_002825.3), respectively. All the variants notations are consistent with HGVS nomenclature using VariantValidator (https://VariantValidator.org). Throughout the paper all NS1 base substitutions, for example the variant NM_002834.5:c.188A>G, is listed as c.188A>G and the resulting amino acid change NP_002825.3:p.(Tyr63Cys) as p.(Tyr63Cys).

The NSEuroNet and COSMIC v.94 data were filtered to remove examples of database errors or internal inconsistencies as well as removing case reports lacking a nucleotide site position or nonmissense variant types. This also included insuring that missense variants reported to cause the closely related *PTPN11* disorder NSML (Noonan Syndrome with multiple lentigines; previously called Leopard syndrome (Allanson & Roberts, 2001 Nov 15 [Updated February 25, 2016]; Tartaglia et al., 2010; Zenker, 2017) were absent.

#### 2.1.1 Source of testes

Testes from anonymous organ donors unaffected by NS1 were purchased from National Disease Research Interchange, Philadelphia, PA (see Declarations). The donors (ages 21, 65, and 68 years) had not been treated with drugs that interfere with normal spermatogenesis and their testes were frozen 10–12 h after death and stored at −80°C.

#### 2.1.2 Testis dissection and DNA isolation

One testis from each unaffected donor was divided into approximately equal pieces (first we cut each testis into six slices, then we further divided each slice into 32 pieces). Details of the testis dissections, DNA isolation and quantitation of the amount of DNA in each piece have been published previously (Choi et al., 2008, 2012; Qin et al., 2007; Shinde et al., 2013; Yoon et al., 2013).

#### 2.1.3 Sequencing library construction

A total of 576 libraries (3 donor's testes × 192 pieces from each testis) were constructed using the SSS Sequencing System (Kinde et al., 2011). One million human genomes were used for each piece. Each strand of each original DNA duplex is uniquely identified by attaching **u** niversal **id** entifiers (UIDs‐random base sequences of 20 nucleotides) to the PCR primers used in the first two amplification rounds (Eboreime et al., 2016). Barcode sequences of length eight nucleotides were also attached to specify each individual testis piece. They were designed such if there is one sequencing mistake in the barcode we could infer the correct barcode, and if there were two sequencing mistakes in the barcode the read would be discarded. The list of barcodes and testis pieces is in Data S1. The pooled libraries were subjected to Illumina 150 bp paired‐end sequencing. Technical details of library preparation are in Methods S1 and S2. The human DNA SSS sequence results can be found using the NCBI BioProject database accession number PRJNA517482 (http://www.ncbi.nlm.nih.gov/bioproject/).

#### 2.1.4 Measuring variant frequencies

Reads with the same UID sequence came from the same original DNA strand. Collections of such reads are called a UID family. If almost all reads in a UID family have the same mutation, this mutation most likely was present in the original DNA molecule as opposed to being a sequencing error. Details of how we implemented these ideas to measure the mutation frequencies are in Methods S3.

**Figure 1:**
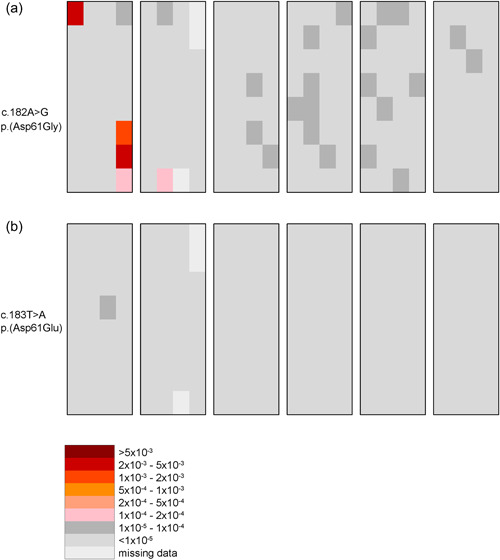
Spatial distribution of variants in the 65‐year‐old testis. The testis was cut into six slices (large rectangles), and each was divided into 32 pieces (checkerboard pattern). Consider the recurrent NS1 variant c.182A>G p.(Asp61Gly) (a) and a control c.183T>A p.(Asp61Glu) (b) sites (in the same testis). The color of each piece represents the variant frequency (the same heat map is used for Figure 2 and Figure S5). (a) Two significant variant clusters were found. The lower portion of slice 1 contains a red piece with a frequency of 4.8 × 10^−3^ and an adjacent orange piece (1.3 × 10^−3^). The second cluster is the red piece in the upper left‐hand corner of the same slice (frequency 4.3 × 10^−3^). (b) All of the testis pieces are colored a shade of gray except for missing data which is the lightest color. There are no significant variant clusters

**Figure 2:**
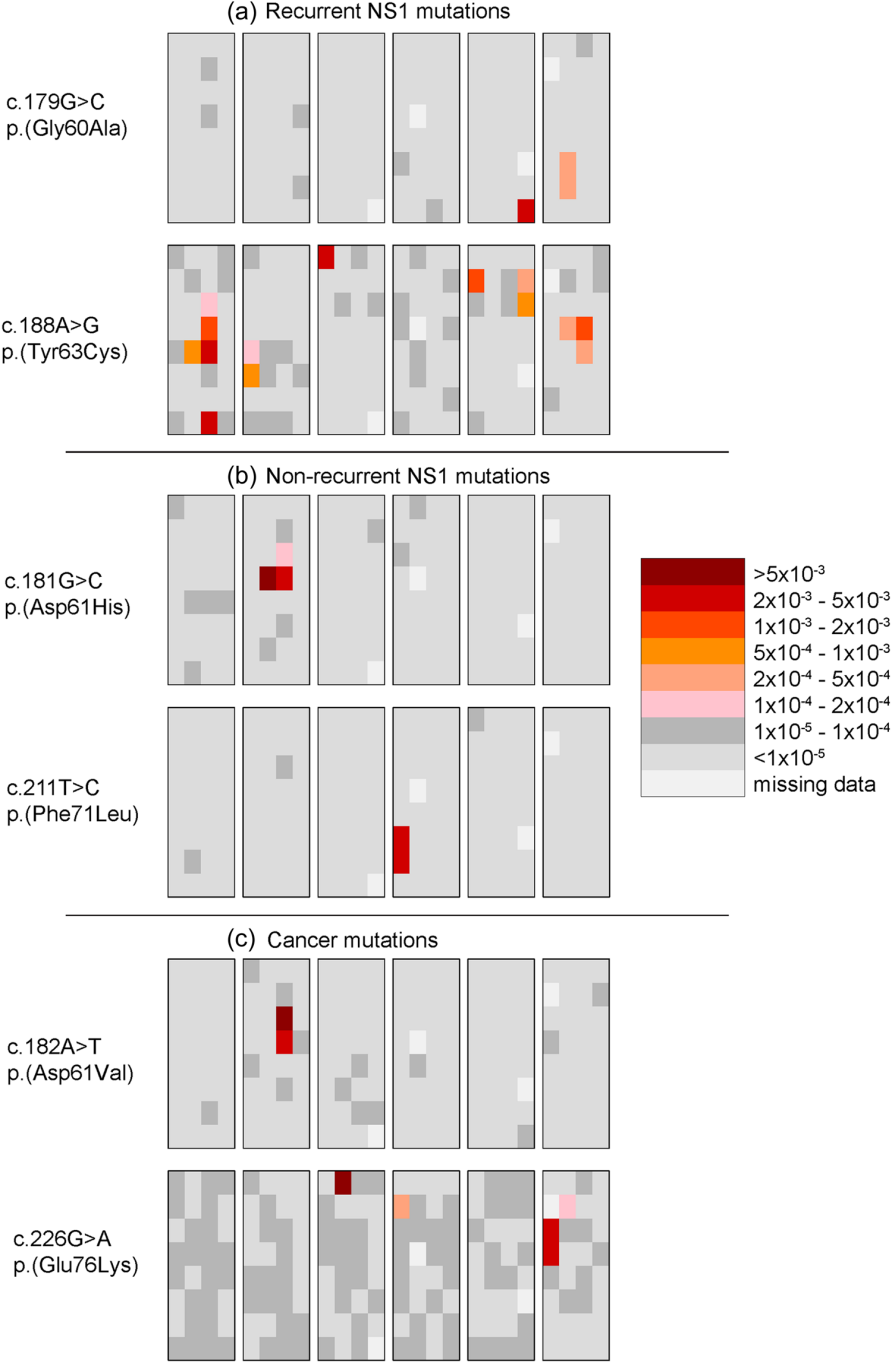
Spatial distributions of six representative significantly clustered variants in the same 68‐year‐old testis. (a) The top two variants are examples of highly recurrent NS1 variants. (b) The middle two variants are examples of rare NS1. (c) The bottom two are examples of S‐O cancer variants

Data on the 576 SSS libraries yielded a total of 667 million reads, which enabled us to confidently determine the sequence for 37 million distinct, original E3 molecules (represented by 37 million UID families). The average number of UID families per testis piece is 64,000. The number of UID families per testis piece is listed in Data S1; Figure S1 shows a histogram of this distribution. To minimize the importance of pieces with a small number of UID families, we designated the seven pieces with fewer than 1000 UID families as missing data (these pieces are colored almost white in the Figures 1 and 2, and Figure S5).

For each testis piece, we determined the variant frequency for each of the 127 consecutive E3 nucleotide sites (108 coding sites and 19 intron sites × 3 possible variants per site = 381) by dividing the number of different UID families with the same base substitution by the total number of different UID families captured from that piece. The precision with which we can measure a mutation frequency per testis piece is one divided by the number of different UID families in that piece. Data S2 lists summary information on all of the E3 nucleotide sites. Data S3 lists detailed information on the variant frequencies in each of the testis pieces.

Previously (Eboreime et al., 2016) we determined the false positive rate of the SSS assay to be less than 3.3 × 10^−6^ for four of the six possible variant types, and 5–6× higher for the remaining two types (G>T/C>A and C>T/G>A) likely due, respectively, to oxidation and deamination damage associated with PCR during the first two SSS cycles. Using the 21‐year‐old testis as a control (this testis does not exhibit mutation clusters), Figure S2 shows that the false positive rate for SSC in this study is similar to our previous work.

#### 2.1.5 Determining significance for observed clusters

Our stem cell proliferation null model is the neutral symmetric hot spot model (Choi et al., 2012). Computer code to simulate this model is available from P.C. upon request. During the growth phase (which models the testes from zygote formation to puberty) there are 30 generations of stem cell symmetric divisions and during the adult phase (which models the testes after puberty) each stem cell divides every 16 days (see Arnheim & Calabrese, 2016) and references therein). The type of stem cell division is random: 50% symmetric divisions (which produce two stem cells) and 50% differentiation (which after a few additional divisions produce sperm). This random balance of the two types of divisions ensures a constant number of stem cells and a continual production of sperm. This model has one free parameter: the mutation rate per cell division (see Arnheim & Calabrese, 2016). See Methods S4 for details of how we used the neutral model to determine significant variants.

#### 2.1.6 NS1 SHP‐2 phosphatase activity dataset

Among the many comparative enzymatic studies on NS1 SHP‐2 variants (Bocchinfuso et al., 2007; Keilhack et al., 2005; Martinelli et al., 2008, 2012; Niihori et al., 2005; Pannone et al., 2017; Tartaglia et al., 2006) we chose two (Keilhack et al., 2005; Tartaglia et al., 2006) that shared very similar methodology in terms of substrates and experimental design to minimize technical differences.

To combine data from different experiments, for each experiment we normalized the relative activity by subtracting the mean relative phosphatase activity for that experiment and then dividing by the standard deviation of the relative activity for that experiment. We then combined this normalized data for the four experiments. We also collected SHP‐2 kinetic data on a number of *PTPN11* sporadic cancer mutations.

#### 2.1.7 Selection models

Earlier studies could explain testis clusters by a germline selection model (Qin et al., 2007; Yoon et al., 2013) where variants arise at the genome average mutation rate per cell division (see Rahbari et al., 2016; Segurel et al., 2014). The symmetric selection model (Yoon et al., 2013) computer code is available from P.C. upon request. It is similar to the neutral symmetric hot spot model (above), except that in the adult phase mutated stem cells (but not wild‐type stem cells) are more likely to divide symmetrically than to differentiate. This model has two free parameters: the mutation rate per cell division and the selection parameter (which quantifies the preference for mutated stem cells to divide symmetrically). See Methods S5 for details on how we used the selection model to predict the incidence rate of NS.

## 3 RESULTS

### 3.1 Analysis of variant E3 testis clusters

Figure 1 shows examples of two E3 nucleotide sites from the same 65‐year‐old testis. Figure 1a depicts c.182A>G p.(Asp61Gly), a NS1 recurrent variant (49 NSEuroNet cases reported). Most of the pieces in this testis are colored light gray indicating a variant frequency less than 10^−5^ (in fact for 85% of these testis pieces no variants were detected at this site), but three of the pieces in this testis are colored red or orange indicating a variant frequency greater than 10^−3^. The highest frequency in all of this testis’ pieces is 4.8 × 10^−3^ (118 distinct mutant c.182A>G UID families among the total of 24,000 different UID families identified in this testis piece). The average c.182A>G variant frequency in this donor's testis, calculated using all the testis pieces is 1.7 × 10^−5^ (175 distinct mutant c.182A>G UID families among the 10 million different UID families identified in this testis). The variants are not uniformly distributed spatially as one would expect if the SSC mutation rate per cell division were elevated at the c.182A site but are clustered indicating a selective advantage for those cells carrying the variant (Maher et al., 2016a; Qin et al., 2007).

In contrast, Figure 1b shows data on the adjacent nucleotide c.183T. We did not identify any of its three possible base substitution mutations in a cancer (Forbes et al., 2017) (COSMIC v94), RASopathy (Zenker, 2017), (NSEuroNet) or ClinVar (Landrum et al., 2018) (https://www.ncbi.nlm.nih.gov/clinvar/) database. All of the testis pieces are as expected and colored a shade of gray with no testis pieces with high variant frequencies. For example, the observed average testis frequency for c.183A>T is 1.0 × 10^−7^ (1 mutant UID family out of the 10 million families detected in this testis), more than two orders of magnitude lower than the testis average for the c.182A>G variant.

#### 3.1.1 Cluster significance

**Figure 3:**
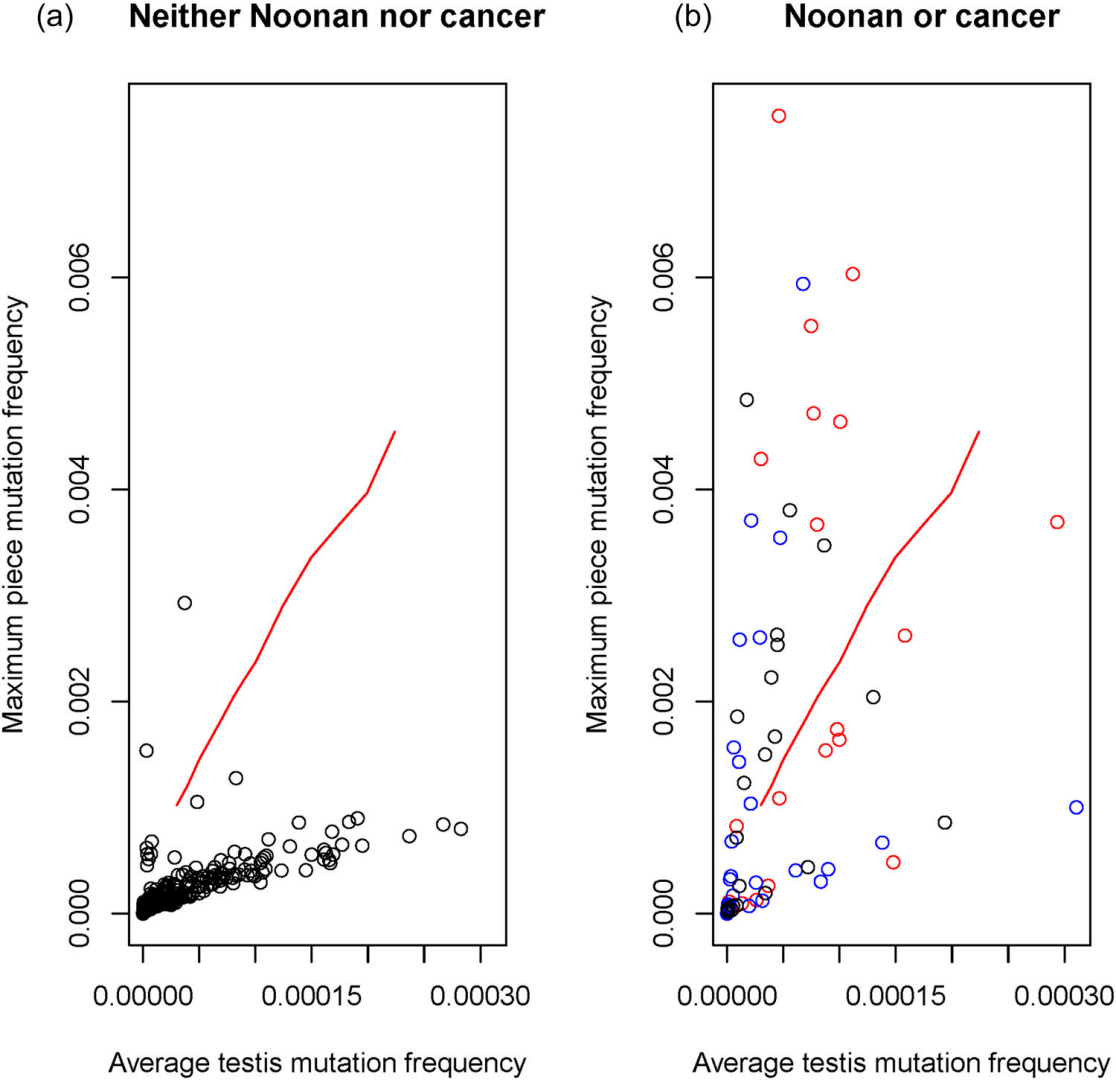
MaxPF v. average testis frequency plots for the two older testes: (a) variants that cause neither NS nor cancer (662 data points including all noncoding, all synonymous, and some nonsynonymous variants) and (b) nonsynonymous variants that cause NS or cancer (100 data points). (b) Partitioned by color: black for recurrent NS1 variants, blue for rare NS1 variants, and red for S‐O cancer variants. Each circle denotes a single variant in one of the older testes (every variant is represented by two circles for the two older testes). The red line is the Bonferroni‐corrected 99th percentile MaxPF as a function of average testis frequency based on simulations of a neutral SSC symmetric division model (see Section 2 for details). Circles above the line are significant variant clusters, and circles below the line are not significant. MaxPF, maximum piece frequency; SSC, safe sequencing system

Data S2 was used to identify significant variant clusters. For each of the testes, every possible variant was characterized by two numbers: the average variant frequency across all the testis pieces and the frequency of the testis piece with the highest mutation frequency (maximum piece frequency [MaxPF]). Figure 3 plots these two statistics for all possible variants in the two older testes (127 nucleotide sites × 3 possible variants × 2 testes = 762 possible variants).

We defined a significant de novo variant cluster as those instances in which the observed MaxPF was exceptionally high relative to the average testis frequency. We defined “exceptionally high” based on computer simulations of the neutral (without selection) germline model of cell proliferation and mutation from zygote formation through adulthood (Choi et al., 2012; Qin et al., 2007; Yoon et al., 2013); see Methods S4 for details. The red line in Figure 3 shows the Bonferroni‐corrected 99th percentile for the MaxPF as a function of average testis frequency according to the neutral model. Variants above the red line are significantly clustered, and variants below the red line are not significantly clustered. This classification is conservative: if any of the variants are not subject to germline selection there is a less than 1% chance that they will be declared to have a significant cluster.

Figure 3 demonstrates that significant variant clusters are atypical. Of the 762 possible E3 variants, 662 (including all noncoding, all synonymous, and some nonsynonymous changes) are not known to cause NS1 nor cancer, while 100 nonsynonymous variants cause either NS1 or sporadic cancer in the population (50 variant sites see Table 1 in each of the two older testes). Figure 3a shows that only two of the 662 variants (0.3%) are significantly clustered (discussed later). In contrast, Figure 3b indicates that 25 out of 100 variants (25%) are significantly clustered (*p* value <2.2 × 10^−16^, Fisher's exact test).

**Table 1:** E3 NS1 and sporadic‐only cancer variant sites

#	Recurrent NS1		Cases	Clusters	#	Rare NS1		Cases	Clusters
1	c.172A>G	p.(Asn58Asp)	20	65	12	c.211T>C	p.(Phe71Leu)	5	(65, 68)
2	c.179G>C	p.(Gly60Ala)	25	68	13	c.214G>C	p.(Ala72Pro)	1	65
	c.181G>A	p.(Asp61Asn)	50		14	c.228G>T	p.(Glu76Asp)	5	68
3	c.182A>G	p.(Asp61Gly)	49	(65,68)		c.236A>C	p.(Gln79Pro)	2	
4	c.184T>G	p.(Tyr62Asp)	42	65					
5	c.188A>G	p.(Tyr63Cys)	110	(65,68)		**S‐O Cancers**			
	c.214G>T	p.(Ala72Ser)	26			c.155C>G	p.(Thr52Ser)	2	
6	c.215C>G	p.(Ala72Gly)	26	68		c.172A>T	p.(Asn58Tyr)	8	
7	c.218C>T	p.(Thr73Ile)	33	68		c.178G>C	p.(Gly60Arg)	7	
	c.228G>C	p.(Glu76Asp)	16			c.179G>T	p.(Gly60Val)	40	
8	c.236A>G	p.(Gln79Arg)	75	68		c.181G>T	p.(Asp61Tyr)	52	
					15	c.182A>T	p.(Asp61Val)	49	68
	**Rare NS1**					c.205G>A	p.(Glu69Lys)	27	
	c.155C>T	p.(Thr52Ile)	2			c.213T>A	p.(Phe71Leu)	9	
9	c.166A>G	p.(Ile56Val)	2	68		c.213T>G	p.(Phe71Leu)	1	
	c.172A>C	p.(Asn58His)	7		16	c.214G>A	p.(Ala72Thr)	45	(65, 68)
	c.173A>G	p.(Asn58Ser)	1		17	c.215C>A	p.(Ala72Asp)	6	68
	c.174C>A	p.(Asn58Lys)	3			c.215C>T	p.(Ala72Val)	64	
	c.174C>G	p.(Asn58Lys)	6			c.220T>A	p.(Leu74Met)	1	
	c.175A>G	p.(Thr59Ala)	1		18	c.226G>A	p.(Glu76Lys)	111	68
10	c.178G>A	p.(Gly60Ser)	2	68		c.226G>C	p.(Glu76Gln)	17	
	c.178G>T	p.(Gly60Cys)	1			c.227A>C	p.(Glu76Ala)	17	
11	c.181G>C	p.(Asp61His)	3	(65,68)	19	c.227A>G	p.(Glu76Gly)	48	68
	c.182A>C	p.(Asp61Ala)	1		20	c.227A>T	p.(Glu76Val)	12	68
	c.184T>A	p.(Tyr62Asn)	1			c.229T>G	p.(Leu77Val)	1	
	c.205G>C	p.(Glu69Gln)	9			c.245T>C	p.(Met82Thr)	1	
	c.206A>T	p.(Glu69Val)	2						
	c.211T>A	p.(Phe71Leu)	1						

*Note*: The cases refer to either the number of NS1 patient reports in the NSEuroNet database or the number of sporadic–only cancer cases (S‐O cancers) in the COSMIC v94 database; for the S‐O cancer variants the number of reported NS1 cases is zero. The 65 and 68 in the Clusters column refers to the older testis donor's age. The numbers (#) 1–20 are used as labels in Figure 4 and Figure S7. Data on E3 cancer variants were obtained from the COSMIC v94 database (see Section 2). The nomenclature for the NS1 and sporadic‐only cancer variants (both nucleotide substitutions and amino acid changes) was validated using VariantValidator.org and are derived from (RefSeq NM_002834.5 and RefSeq NP_002825.3).

**Figure 4:**
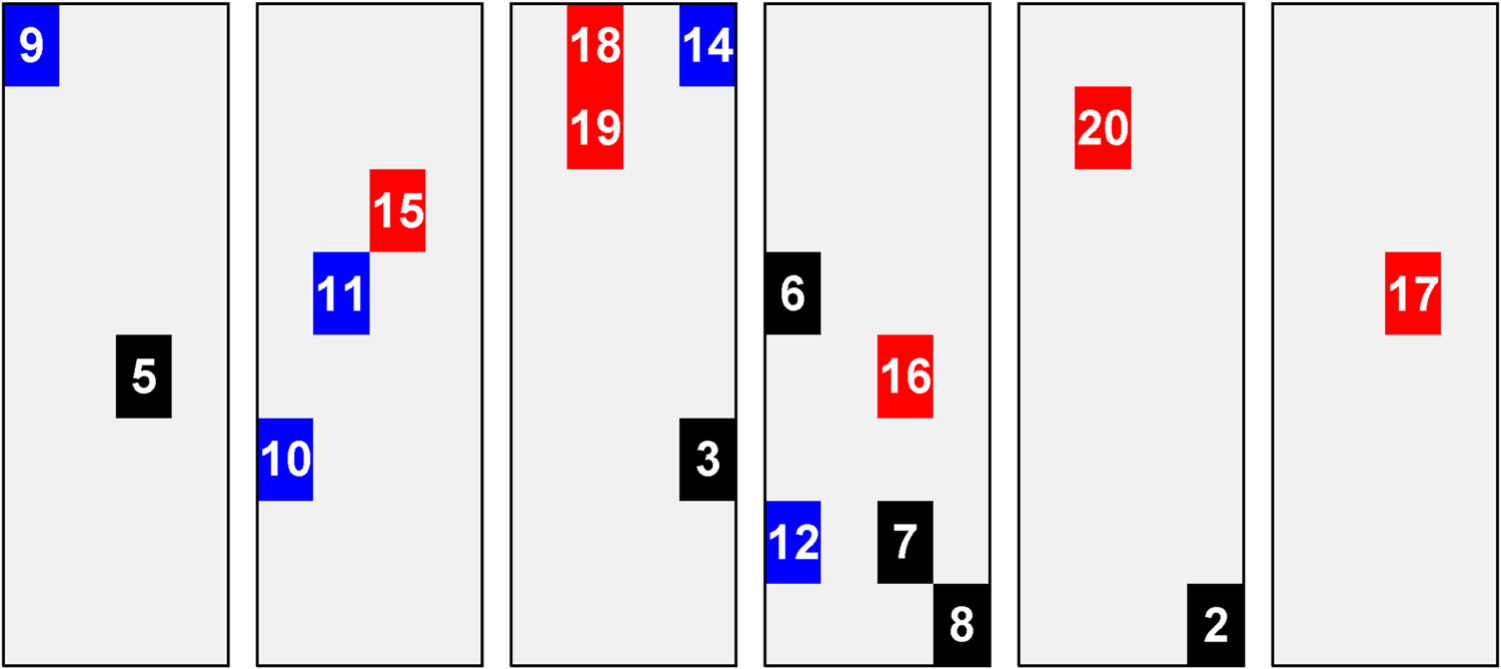
All 17 significant clusters in the same 68‐year‐old testis. For each variant with a significant cluster, we have chosen the one testis piece with the maximum frequency for that variant. The numbers in the testis pieces correspond to the variant labels in Table 1. Color indicates variant type: recurrent NS1 variants—black, rare NS1 variants—blue, and S‐O cancer variants—red

The 21‐year‐old testis, unlike the two older testes, had no variants with high MaxPFs (Figure S3), confirming that variant clusters grow in the adult because the chance of a de novo variant arising during early development is unlikely (reviewed in Arnheim & Calabrese, 2016; Goriely & Wilkie, 2012). Also as expected, the average testis variant frequencies were higher for C>T/G>A and G>T/C>A types compared to the four other mutations types (Figure S4), due to the higher background of the SSS assay for these two types (Eboreime et al., 2016; Kinde et al., 2011).

The two testes from the older unaffected donors captured 47% of the 30 known E3 NS1 variant sites (14/30, Table 1). One testis had significant clonal testis clusters from 11 different E3 NS1 sites. Figure S5 shows the spatial distribution pattern for all E3 variants that are significantly clustered. Further, the reader can create spatial distribution figures for any of the possible E3 variants (127 nucleotide sites × 3 possible variants × 3 testes = 1143 possible variants) by downloading Data S3 which has the raw variant frequency data for all of the testes pieces. The Note S1 has the instructions and Code S1 has the R‐code to make the figures.

#### 3.1.2 NS1 recurrence variation in E3

A total of 11 of the 30 E3 NS1 variant sites are repeatedly reported in NSEuroNet; for these 11 sites the average number of cases per site is 43.0 (range: 16–110, Table 1). Eight of these 11 sites formed significant selection‐driven testis clusters in the two older donors (two of these variant sites were clustered in both older testes). Figure 2a shows the spatial distribution of two such representative variants with significant clusters in the same 68‐year‐old testis: c.179G>C p.(Gly60Ala), (25 reported cases) forms one cluster, and c.188A>G p.(Tyr63Cys) (110 reported cases) forms several clusters.

E3 also contains 19 NS1 variant sites that are rarely reported in the NS1 population (NSEuroNet); the average number of cases for these sites is 2.9 (range: 1–9, Table 1). Six of these 19 sites formed significant clusters (two variant sites were clustered in both older testes). Figure 2b shows two such representative NS1 variants with significant clusters within the same 68‐year‐old testis: c.181G>C p. (Asp61His) and c.211T>C p.(Phe71Leu), three and five cases reported, respectively.

The chance that a man passes a new mutation to his child is presumably the average testis frequency for this variant at the age of fatherhood. Figure S6 shows that for the 30 E3 NS1 variant sites, there is no correlation (*p* value 0.38, correlation coefficient 0.12) between the average testis variant frequency and the number of reported NS1 cases.

#### 3.1.3 E3 cancer variants

*PTPN11* contains E3 nucleotide sites that can undergo somatic missense mutations causing sporadic cancers in the general population (such as juvenile myelomonocytic leukemia, myelodysplastic syndrome, and others [Forbes et al., 2017; Kratz et al., 2007; Mohi & Neel, 2007; Tartaglia et al., 2003]). Data S2 includes 35 E3 H&LN nucleotide sites and the numbers of sporadic cancers currently reported in the COSMIC (v94) database (after appropriate filtration, see Section 2). We identified 20 of these 35 nucleotide sites as “sporadic‐only” (S‐O, Table 1) as they have not been found to be associated with the NS1 patient population (Forbes et al., 2017; Tartaglia et al., 2003; Zenker, 2017).

We observed that six of the 20 S‐O sites formed significant testis clusters (one variant site was clustered in both older testes). Figure 2c shows the testis distribution of two such representative clusters: c.182A>T p.Asp61Val), 0 NS1 cases, 49 sporadic cancer cases and c.226G>A p.(Glu76Lys), 0 NS1 cases, 111 sporadic cancer cases. Interestingly, among the four remaining clustered E3 S‐O testis variants, three c.214G>A p.(Ala72Thr), c.227A>T p.(Glu76Val), and c.227A>G p.(Glu76Gly) were identified following pregnancy termination and DNA analysis in early fetuses that underwent ultrasound examination in search of possible signs of NS or related disorders (Croonen et al., 2013; Mason‐Suares et al., 2017).

#### 3.1.4 Human testes can be multimosaics of E3 NS1 and sporadic cancer variants

To emphasize that different E3 variant sites can form clusters that grow independently in the same testes, Figure 4 shows the spatial location of all of the significant clusters in the same testis from the 68 years old. For each variant with a significant cancer or NS1 cluster, we have indicated the one testis piece with the MaxPF. Similarly, Figure S7 shows the spatial location of all the significant E3 clusters in the 65‐year‐old testis (at four NS1 recurrent sites, three rarely observed NS1 sites, and one S‐O cancer site). As men age, their germlines become mosaic for different RAMP/PAE variants due to different de novo PTPN11 variants undergoing clonal expansion (also see Section 4).

#### 3.1.5 Hitchhiking E3 variant

Of the 660 possible E3 variants not found to cause either NS1 or cancer, only two are significantly clustered. One is a synonymous variant c.216C>T p.(Ala72Ala) in the 68‐year‐old donor. A de novo synonymous variant arising in a single SSC already carrying a de novo nonsynonymous mutation with a selective advantage could form a significant testis cluster containing both variants (Goriely & Wilkie, 2012). Note that the S‐O cancer variant c.215C>A p.(Ala72Asp) shows the same cluster colors in the same slice and position (Figure S5, slice 2, adjacent pieces colored red and orange in the left‐most column). Further, in these two testis pieces, all of the reads with one variant also have the other variant. The synonymous variant has “hitchhiked” with the cancer variant. Figure S5 also shows that the cancer variant has additional significant clusters in slices 5 (two adjacent pieces colored red) and 6 (one piece colored brown) that the synonymous variant does not share.

#### 3.1.6 Puzzling *presumptive* E3 variant

The NS1 p.(Lys55) codon within the 97 amino acid N‐terminal Src homology 2 domain (N‐SH2) plays a critical role in SHP‐2 function (Neel et al., 2003). We searched NSEuroNet, ClinVar, COSMIC, and DECIPHER (https://www.deciphergenomics.org/) databases for the *presumptive* NM_002834.5:c.A>G (*PTPN11* 12:112450344A>G) variant c.164A>G p.(Lys55Arg) without finding it. Importantly, the Genome Aggregation Database (gnomAD, v2.1.1, https://gnomad.broadinstitute.org/) did not contain p.(Lys55Arg) among a total of 250,000 sequenced alleles that included approximately 109,000 sequenced “control” alleles.

Three in silico prediction protocols assessed p.(Lys55Arg)'s likely functional impact. PolyPhen‐2 (Adzhubei et al., 2010) judged it to be benign and PROVEAN (Choi et al., 2012) concluded it was neutral. However, PON‐P2's (Niroula & Vihinen, 2015) “Probability of Pathogenicity” value was 0.779 (SE 0.097) but the Prediction was “unknown.”

We aligned human SHP‐2 with fish, amphibian, reptile, bird and primate homologs (Figure S8. The p.(Lys55) codon was conserved among all the species we examined. The alignment of human SHP‐2 with the zebrafish (Danio rerio) homolog ptpn11a (Bonetti et al., 2014) showed (Figure S9) them to be 91% identical, (the N‐SH2 domain, 100%) even though fish and humans last had a common ancestor approximately 400 mya (Lu et al., 2017). The details of these protocols and inconsistency of results are discussed in Methods S6.

#### 3.1.7 NS1 variant recurrence throughout *PTPN11*

We determined (1) whether the recurrence rate for cases at any particular NS1 variant site depend on the amino acid substitutions effect on SHP‐2's phosphatase activity and (2) if germline selection of many *PTPN11* NS1 variants contribute to the high incidence of NS in the population. To study these issues we had to use data from all of *PTPN11*, not just E3.

To justify combining our E3 data with other NS1 *PNPN11* variants we first demonstrated that the ratio of transitions to transversions (Ti/Tv) in mutations causing NS1 is the same in E3 as in the rest of *PTPN11* (Chi‐squared test *p* value 0.20, see Figure S10). Similarly, comparison of the number of reported NS1 cases at each NS1 variant site (Figure S11) in these two *PTPN11* regions lacked sufficient evidence to reject the null hypothesis that the distribution of case numbers is the same in both regions (Wilcox Mann–Whitney test, *p* value 0.06).

#### 3.1.8 Correlation of NS1 variant case recurrence with SHP‐2 activity

**Figure 5:**
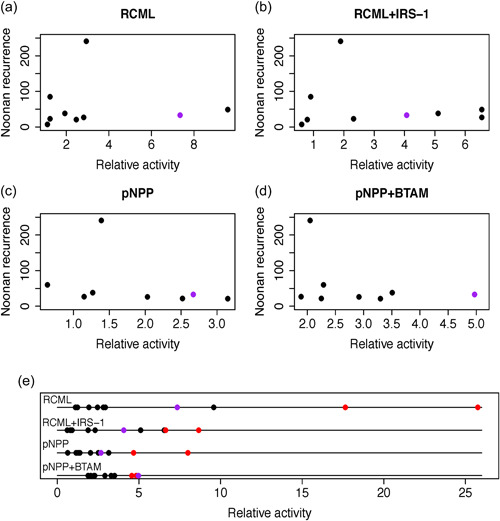
Correlation between NS recurrence level and phosphatase activity. No significant correlation exists between the number of reported NS1 cases at recurrent sites and the levels of mutant phosphatase activity (relative to wild type SHP‐2) for four different substrate conditions [(a)–(d), also see Figure S12]. Subplot (e) shows relative levels of mutant phosphatase activity are higher for *PTPN11* sporadic cancer variants (red circles: p.(Asp61Tyr), p.(Ala72Val) and p.(Glu76Lys)) than recurrent NS1 mutants (black circles, *p* value <10^−5^, see Section 2). *Note*: variant p.(Thr73Ile) is colored purple because it is both a significant recurrent NS1 variant (33 cases) and a sporadic blood cancer variant (20 sporadic cases). RCML and pNPP are basal phosphatase substrates; BTAM and IRS‐1 are N‐SH2 domain binding tyrosine phosphopeptides that can alter SHP‐2 enzyme activity (see Keilhack et al., 2005; Tartaglia et al., 2006)

We originally speculated the varying NS1 recurrence levels might be correlated with the extent of SHP‐2 tyrosine phosphatase hyperactivation characteristic of the different NS1 amino acid changes. Since higher levels of hyperactivation are generally associated with poor developmental outcomes (Kratz, 2005; Lee et al., 2009; Mohi & Neel, 2007; Strullu et al., 2014; Tartaglia et al., 2001, 2003, 2006) we predicted that more recurrent variants might have lower levels of SHP‐2 hyperactivation than the rare variants. The two datasets we choose (Keilhack et al., 2005; Tartaglia et al., 2006) sampled multiple regions of *PTPN11* (including several E3 NS1 variant sites) and shared very similar substrates and experimental designs. Each data set used a basal substrate alone or the basal substrate plus a peptide containing a phosphotyrosine known to bind SHP‐2; the two datasets used different basal substrates and phosphopeptides. We evaluated 12 SHP‐2 NS1 variant sites (four from E3) ranging from 7 to 241 reported cases/site; average cases/site = 53. Figure 5a–d shows the scatter plots for the two datasets (see Figure S12). We found no significant correlation between the levels of mutant phosphatase activity (relative to wild type SHP‐2) and the number of reported recurrent NS1 cases (*p* value 0.24, correlation −0.21). In addition, SHP‐2 phosphatase activity of the three tested *PTPN11* S‐O cancer variants, shown in Figure 5e, are significantly higher than the recurrent NS1 variants (*p* value <3 × 10^−5^, explained in Methods S7) confirming earlier work (Bocchinfuso et al., 2007; Keilhack et al., 2005; Martinelli et al., 2008, 2012; Niihori et al., 2005; Pannone et al., 2017; Tartaglia et al., 2006).

#### 3.1.9 The role of selection in NS birth incidence

Earlier studies could explain testis clusters by a germline selection model (Qin et al., 2007; Yoon et al., 2013) where variants arise at the human genome average mutation rate per cell division (see Rahbari et al., 2016; Segurel et al., 2014). In the selection model, the mutated SSC, unlike the wild type SSC, are slightly more likely to self‐renew than to differentiate, enabling variant SSS clusters to grow. This stochastic selection model is consistent with the E3 testis data. Simulations corresponding to the ages of the older donors match the data both (1) in terms of the fraction of simulations with significant clusters, and (2) for those simulations with significant clusters, both the MaxPF and the average testis frequency are similar to the frequencies observed in the testes. Also, simulations corresponding to the age of the 21‐year‐old donor do not produce significant clusters, likely due to the relatively small number of SSC self‐renewal divisions between puberty and their age at testis donation. Simulations of the selection model predict the birth rate for NS to be 8.8 × 10^−4^ which is in good agreement with the observed NS birth rate estimate range (4 × 10^−4^–10^−3^). Details in Methods S5.

#### 3.1.10 Comparison with previously studied RAMP/PAE mutations

**Figure 6:**
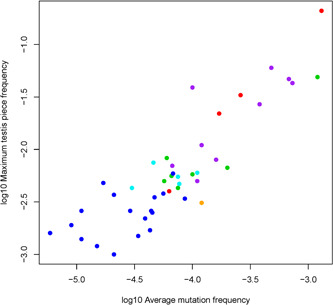
Log MaxPF versus log average testis frequency for different significant RAMP/PAE variant clusters. The E3 variants from this paper are shown in the dark blue for NS1 and light blue for S‐O cancers. The remaining data come from our earlier studies on RTK variants causing Apert syndrome (MIM**#** 101200), Achondroplasia (MIM**#** 100800), or Multiple Endocrine Neoplasia Type 2B (MIM**#** 162300) (Choi et al., 2008, 2012; Shinde et al., 2013; Yoon et al., 2013). The MEN2B variant (NM_020975.6(RET):c.2753T>C (p.Met918Thr)) from seven donors is colored green, the achondroplasia variant (NM_000142.5(FGFR3):c.1138G>A (p.Gly380Arg)) from one donor is colored orange and the Apert variants (NM_000141.5(FGFR2):c.755C>G (p.Ser252Trp)) and (NM_000141.5(FGFR2):c.758C>G (p.Pro253Arg)) from four donors provided data on both variants and a fifth donor provided data on only one of the variants (for a total of nine purple data points). The most frequent NS1 variant p.(Asn308Asp) in exon 8 from four donors is shown in red. See Methods S8 for additional details

RAMP/PAE mutations in receptor tyrosine kinases (RTK) have been shown to have significant mutation clusters caused by germline selection (reviewed in Arnheim & Calabrese, 2016; Goriely & Wilkie, 2012). Figure 6 shows a Log–Log plot of the MaxPF vs. average testis frequency for the RAMP/PAE clusters of E3 SHP‐2 and the RTK variants in *FGFR2*, *FGFR3*, or *RET* (for details see the Figure Legend, note: these earlier papers had varying numbers of testis donors). In Methods S8, we give a mathematical explanation for the data points lying roughly on a straight line. The significant E3 variants (blue) have both lower MaxPF and smaller average testis frequencies than the previously studied RAMP/PAE variants (other colors). If we make the simplifying assumption that the 110 NS1 variants (other than p.Asn308Asp in exon 8) contribute equally then the E3 variants have apparent substitution rates approximately 25–50 times greater than the known genome average (details in Methods S8). The variants colored green, purple, and orange in Figure 6 have apparent substitution rates several hundred times greater than the genome average (Choi et al., 2008, 2012; Giannoulatou et al., 2013; Qin et al., 2007; Shinde et al., 2013). The p.Asn308Asp variant colored red has an apparent nucleotide substitution rate 2,400 times greater than the genome average (Yoon et al., 2013). So the order of the different colored mutations in Figure 6 roughly corresponds to the expected order based on disease incidences.

## 4 DISCUSSION

Genetic transmission of *PTPN11* germline NS1 and S‐O cancer variants produce vastly different consequences ranging from fetal death to live‐born moderately affected individuals with NS1. These causal variants change SHP‐2 protein conformation, both locally and at a distance, due to the specific amino acid substitution and its particular location in the protein's three‐dimensional structure initiating a gain of function phenotype (Darian et al., 2011; Hof et al., 1998; Martinelli et al., 2012; Tartaglia et al., 2006). SHP‐2 is autoinhibited in a basal state by intramolecular interactions between the N‐SH2 domain and catalytic protein domains and acts as an intramolecular switch to control SHP‐2 tyrosine phosphatase activity. Extensive evidence documents how different *PTPN11* E3 variants alter the sensitivity of SHP‐2's switch leading to significant variations in the increase in SHP‐2 tyrosine phosphatase activity (Kratz, 2005; Lee et al., 2009; Mohi & Neel, 2007; Strullu et al., 2014; Tartaglia et al., 2001, 2003, 2006; Zhu et al., 2020). The consequences directly, or indirectly, influence SHP‐2's interaction with tyrosine‐phosphorylated SHP‐2 ligands, docking proteins and target proteins dysregulating the RAS/MAPK pathway (Dance et al., 2008; Edwards et al., 2014; Fragale et al., 2004; Müller et al., 2013; Neel et al., 2003; Pannone et al., 2017; Tao et al., 2021; Zhu et al., 2020).

The pathogenic consequences of increased RAS/MAPK signaling in NS1 include pathological alterations in specific cell types during development. In vivo/vitro mouse studies on the role of NS1 variants leading to heart conditions and hydrocephalus have examined pathways downstream of RAS/MAPK responsible for the pathogenic effects in the NS1 target tissues and showed variation depending on the tissue and the specific NS1 variant (Araki et al., 2004; Krenz et al., 2008; Zheng et al., 2018). Interestingly, genetic data support a role for Stat3 in mouse SSC proliferation (He et al., 2013; Kaucher et al., 2012; Oatley et al., 2010). Biochemical studies on human and mouse NS1 variants show both Stat3 (MGI:103038) or STAT3 (HGNC:11364) activity is reduced by SHP‐2's dephosphorylation of critical phosphotyrosines in different somatic tissues (Zhang et al., 2009; Zheng et al., 2018).

Germline selection of mutant SSC is expected to be defined primarily by the transcriptional and proteomic status of these testis cells. The nature of this profile in the germline may have similarities to somatic tissues that also form cell clusters carrying oncogenic variants without histological changes (Martincorena et al., 2018; Martincorena, 2019). Many common cancer driver genes show marked tissue specificity (Haigis et al., 2019; Schneider et al., 2017) and SHP‐2 has been classified primarily as an oncogenic driver gene for sporadic hematopoietic and lymphoid neoplasms (Bailey et al., 2018; Chan & Feng, 2007). Tissue specificity may explain why SHP‐2 and other RAMP/PAE variants have not been found associated with any testicular cancers (COSMIC v94 [Forbes et al., 2017]) and are even absent in a rare testis cancer typically found only in elderly men (spermatocytic seminomas (Goriely et al., 2009) where it most likely would have been expected).

The population of SSC in a man's testis presumably share the same molecular features but the potential for testis cluster formation after the acquisition of a de novo RAMP/PAE variant may take distinct pathways due to each particular amino acid substitution's functional impact on the SSC. The data in Figure 6 may be an example of this kind of variation.

We also examined the idea that the level of population recurrence of different NS1 variants might be expected to be significantly correlated with SHP‐2 hyperactivity. It is generally accepted that mutants with the highest levels of SHP‐2 activity are usually associated with the most serious clinical outcomes (Kratz, 2005; Lee et al., 2009; Mohi & Neel, 2007; Strullu et al., 2014; Tartaglia et al., 2001, 2003, 2006). This suggests NS1 variants leading to more limited developmental potential and fewer successful pregnancies would lower the likelihood of genetic transmission and contribute to lower recurrence rates in the next generation. We used data on a sample of SHP‐2 variants where both tyrosine phosphatase activity and birth recurrence rates were established. We found no evidence (Figure 5) for a correlation between SHP‐2 activity and NS1 case recurrence rate and no correlation between the average testis variant frequency and the number of reported NS1 cases among the 30 E3 NS1 variants (Figure S6).

A more likely explanation for NS1 variant recurrence differences is that selection may depend less on absolute tyrosine phosphatase activity and more on alteration of SHP‐2's interaction with tyrosine‐phosphorylated SHP‐2 ligands, docking proteins and target proteins as mediated by conformational changes induced by different NS1 amino acid substitutions (Fragale et al., 2004; Kratz, 2005; Lee et al., 2009; Mohi & Neel, 2007; Strullu et al., 2014; Tartaglia et al., 2001; Tartaglia et al., 2003; Tartaglia et al., 2006). Additional biochemical data on the downstream effects of different SHP‐2 NS1 variant types is needed to confirm this idea.

*PTPN11* E3 contains 35 filtered COSMIC v94 sporadic cancer nucleotide sites contributing to sporadic blood cell cancers. Among these are sites each capable of providing two distinct outcomes after a de novo mutation. If the nucleotide substitution at the site occurs in a somatic lineage (e.g., myeloid blood cells), a *PTPN11*‐driven sporadic blood cell cancer case may result in the general population whereas if the same nucleotide substitution arises in an SSC the variant may be transmitted to the next generation (for examples, see Data S2).

We call 20 of the 35 E3 sporadic blood cancer sites S‐O as they were previously classified as unassociated with NS1 and were recognized as having SHP‐2 tyrosine phosphatase hyper‐activation levels that would likely prevent germline transmission due to early developmental failure (Lee et al., 2009; Niihori et al., 2005; Tartaglia et al., 2003, 2006). Recent ultrasound studies combined with molecular analysis (Croonen et al., 2013; Hakami et al., 2016; Lee et al., 2009; Mason‐Suares et al., 2017) shows strong support for germline transmission of some of these S‐O cancer variants. The three S‐O variants in the early‐stage fetuses discussed above (Croonen et al., 2013; Mason‐Suares et al., 2017) are by definition compatible with normal spermatogenesis and fertilization. They survived through 19 weeks of pregnancy that inevitably lead to abnormal development.

*PTPN11* codon Lys55 has not changed over the last 400 million years and has yet to be reported in human variation databases. The *presumptive* p.(Lys55Arg) variant cluster we found requires studies on the functional properties of a synthetic version of this *presumptive* variant to decide whether it is a rare but unlikely neutral variant cluster (see Choi et al., 2012; Yoon et al., 2013) or a highly deleterious substitution incompatible with development yet capable of initiating clonal expansion in SSC.

Early ultrasound screening has also identified fetuses carrying recurrent and rare NS1 variants as well as germline derived sporadic cancer variants that might have been recognized as NS1 cases if they had further completed development (Croonen et al., 2013; Hakami et al., 2016; Leach et al., 2019; Lee et al., 2009; Mason‐Suares et al., 2017). Additional studies are likely to provide more information given its importance to prenatal diagnosis.

Although difficult to measure, about 40%–60% of human embryos may be lost between fertilization and birth (Jarvis, 2016) due to genetic (usually chromosomal abnormalities), environmental or some medical conditions (Larsen et al., 2013). Any deleterious de novo gain‐of‐function variants that contribute to this type of loss would have a far greater negative reproductive impact if they also conferred a germline selective advantage to the SSC in the testis. As men age a disproportionately larger percentage of sperm would carry the mutant (compared to sperm without this advantage). Additional studies are needed to better understand the overall impact of germline selection on reproduction.

Extrapolating the effect of germline selection to 110 known filtered NS1 sites (regardless of recurrence rate) allowed us to predict a NS birth rate that is within the generally accepted range (1/1000–1/2500 births) (Allanson & Roberts, 2001 Nov 15 [Updated February 25, 2016]). These results provide the first quantitative data supporting the hypothesis (Yoon et al., 2013) that selection acting on many different RAMP/PAE variant sites in one gene may be a major contributor to the high birth rate of NS and possibly applicable to other genetic conditions (Arnheim & Calabrese, 2016; Choi et al., 2008, 2012; Maher et al., 2016a, 2018).

Finally, we were surprised that the E3 testis clusters formed by different *PTPN11* variants have very similar anatomical distributions, average testis variant frequencies and MaxPFs (see Figure 6) regardless of their NS1 case recurrence rate or contribution to sporadic cancers in the general population. A greater understanding of the transcriptional and protein environment of wild type and mutant SSC could be achieved by single cell RNAseq studies coupled with single cell protein methods (reviewed in Guo et al., 2020; Zhu et al., 2020) and would have a profound effect on elucidating germline selection mechanisms in detail.

## WEB RESOURCES

ClinVar (aggregates information about genomic variation and its relationship to human health (https://www.ncbi.nlm.nih.gov/clinvar/)

COSMIC v94, Catalogue of Somatic Variants In Cancer, https://cancer.sanger.ac.uk/

DECIPHER, DatabasE of genomiC varIation and Phenotype in Humans using Ensembl Resources (https://www.deciphergenomics.org/)

E!Ensembl https://ensembl.org/index.html

European Network on Noonan syndrome and related disorders database https://nseuronet.com

gnomAD (v2.1.1), https://gnomad.broadinstitute.org/

HGVS nomenclature using VariantValidator (https://VariantValidator.org)

Mendelian Inheritance in Man (MIM#) https://www.omim.org

NCBI BioProject database (http://www.ncbi.nlm.nih.gov/bioproject/

PolyPhen‐2 (http://genetics.bwh.harvard.edu/pph2/dbsearch.shtml),

PON‐P2 Protein Variation Effect Analyzer (http://structure.bmc.lu.se/PON-P2/)

PROVEAN (http://provean.jcvi.org/index.php),

PTPN11 https://www.ncbi.nlm.nih.gov/protein/33356177

SHP‐2 https://www.uniprot.org/uniprot/Q06124

STAT3 UniProtKB ‐ P40763 (STAT3_HUMAN) https://www.uniprot.org/uniprot/P40763

## AUTHOR CONTRIBUTION

Experiments were designed by J.E., P.C and N.A and carried out by J.E. using methods developed by S‐R.Y, S‐K.C. and J.E. Both A.S and V.K contributed to SHP‐2 protein structure‐function analysis. P.C was responsible for sequencing data and statistical analyses. The manuscript was written by N.A. and P.C. who both had overall supervision and conceived of the project.

## CONFLICTS OF INTEREST

The authors declare no conflicts of interest.

## Supporting information

Supporting information.

Supporting information.

Supporting information.

Supporting information.

## Data Availability

Dataset(s) supporting the conclusions of this article are included within the article, its Supporting Information and the NCBI BioProject database (see Section 2). Information supporting the conclusions of this article are included within the article, its Supporting Information and the NCBI BioProject database (accession number PRJNA517482). Our unpublished code is found in the Supporting information. Codes used in our previously published papers are available from P.C. on request.
