# Supplementary material for: Germline selection of PTPN11 (HGNC:9644) variants make a major contribution to both Noonan syndrome's high birth rate and the transmission of sporadic cancer variants resulting in fetal abnormality
Source: Hum Mutat. 2022 Nov 24;43(12):2205–21. doi: 10.1002/humu.24493 (PMC10099774; doi:10.1002/humu.24493)
Supplement: Supplementary file 1 — Supporting information. [file HUMU-43-2205-s002.pdf]

Supporting information Eboreime et al. (humu-2022-0107)

Germline selection of *PTPN11* variants makes a major contribution [illegible] both Noonan syndrome's high birth rate and [illegible] fetal [illegible]

Jordan Eboreime, Soo-Kyung Choi, Song-Ro Yoon, Anastasiia Sadybekov, Vsevolod Katritch, Peter Calabrese, and Norman Arnheim

Corresponding author: Norman Arnheim; arnheim@usc.edu

Content

Supp_Mat Note 1

Supp_Mat Code 1

Supp. Figures S1-S12

Supp_Mat Methods 1-8

Supp_Mat Data 1-3

Supp_Mat References

Supp_Mat Note 1 Instructions to create images of the spatial distribution of variants in the testes similar to Figure 1, Figure ʜ,   and Figure Sp

Instructions

1. Download the file Supp_Mat Data3.csv. This file contains the variant frequency data for all possible variants at all studied nucleotide positions for each testis piece in the three testes. The format is explained below these instructions*.
2. Cut and paste the text in Supp_Mat Code 1 and save it in a file named SCode.r. This is the R code to make the images.
3. If you do not already use the free statistical computing and graphics program R, download it from: https://www.r-project.org
4. In R, change the working directory to where you have placed the files Supp_Mat Data3.csv and SCode.r.
5. In R, type:

source("SCode.r")

6. You are now ready to make images. For example, for the 65 year old to show the c.182A>G variant, in R type:

show(x60649,182,"G",1000)

a) The first argument (x60649) is the identity of the testis. There are three possibilities: x60649 (65 year old), x60891 (68 year old), or x63878 (21 year old).
b) The second argument (182) is the nucleotide position. The possible positions are 119 to 245.
c) The third argument ("G") is the base of the variant. The possible bases are "A", "C", "G", or "T".
d) The fourth argument (1000) is to minimize the importance of any testis piece with a small amount of data. For each testis piece, there is a number in the numerator and a number in the denominator to calculate the variant frequency. If the number in the denominator is less than the fourth argument (in this case 1000, but the reader can use any threshold they like) that particular testis piece will be colored off-white.

* Explanation of the format of Supp_Mat Data3.csv. Columns 1 to 129 are for testis x60649 (65 year old), columns 130 to 258 are for testis x60891 (68 year old), and columns 259 to 387 are for testis x63878 (21 year old). For each of these three testis blocks, the first two columns give the location of the testis piece: slice (1 to 6) and piece (1 to 32). The first row indicates the identity of the testis. The second row gives the nucleotide position (119 to 245). The third row gives the base of the reference sequence (1=A, 2=C, 3=G, 4=T). The remaining rows give the variant frequency information. For each testis piece, there are five consecutive rows: the top row gives the number of distinct molecules studied in that testis piece (distinct means they are not PCR copies but molecules present in the original sample before amplification, this number is the same as the number of UID families; the reason this number varies between nucleotide positions is due to sequence quality considerations), the next row gives the number of distinct molecules with an A variant (at the nucleotide position in that testis piece), the next row gives the number of distinct molecules with a C variant (at the nucleotide position in that testis piece), the next row gives the number of distinct molecules with a G variant (at the nucleotide position in that testis piece), and the last row gives the number of distinct molecules with a T variant (at the nucleotide position in that testis piece). There are similar sets of five consecutive rows for all testis pieces.

Sub_Mat Code 1 R-code to create images of the spatial distribution of variants in the testes

```
# START SCODE
# get testis data

fname = "humu24493-sup-0004-supp_mat_data3.csv";
x3 = read.csv(fname,skip=2,header=F);
x60649 = x3[,1:129];   # 65 year old
x60891 = x3[,130:258]; # 68 year old
x63878 = x3[,259:387]; # 21 year old

# show function shows testis data
# Inputs:
# x = testis data (either x60649, x60891, or x63878)
# tpos = nucleotide position (from 119 to 245)
# nall = new allele (either "A", "C", "G", or "T")
# th = threshold (pieces with little data colored off-white)

show <- function(x,pos,nall,th) {
  mnfreq = 0;
  keeptot = 0;
  topfreq = 0;
  allfreq = 1:192;
  acnt = 0;

  mybreaks = c(-20,0,10,100,200,500,1000,2000,5000,10000) - 0.5;
  mycolors = c("gray93","gray85","gray70","pink","lightsalmon");
  mycolors = c(mycolors,"darkorange","orangered","red3","darkred");


  nslices = 6;
  xxx = array(0*(1:(8*4*6)),c(8,4,6));
  # start with white, some coords never assigned
  for (i in 1:6) {
    for (j in 1:4) {
      for (k in 1:8) {
        xxx[k,j,i] = -10;
      }
    }
  }

  # the fun
  zz = acrosspc(x,pos);
  coord = getcoords(x);
```

```
  for (i in 1:length(coord$slice)) {
    xy = getxy(coord$piece[i]);
    if (zz$tot[i] < th) { freq = -10; }
    else {
      if (nall == "A") { freq = zz$aa[i]/zz$tot[i]; }
      if (nall == "C") { freq = zz$cc[i]/zz$tot[i]; }
      if (nall == "G") { freq = zz$gg[i]/zz$tot[i]; }
      if (nall == "T") { freq = zz$tt[i]/zz$tot[i]; }
      freq = freq*1000000; # units of mutants per million molecules
    }
    xxx[xy$y,xy$x,coord$slice[i]] = freq;
  }

  par(mfrow=c(1,nslices),mar=c(15,.5,15,.5));
  for (i in 1:nslices) {
   image(t(xxx[8:1,,i]),breaks=mybreaks,col=mycolors,axes=FALSE);
   box();
  }
}

# showmap function shows heatmap colors

showmap <- function() {
  mybreaks = c(-20,0,10,100,200,500,1000,2000,5000,10000) - 0.5;
  mycolors = c("gray93","gray85","gray70","pink","lightsalmon");
  mycolors = c(mycolors,"darkorange","orangered","red3","darkred");

  x = matrix(nrow=9,ncol=1);
  x[,1] = c(-10,5,50,150,400,750,1500,3000,8000);
  image(t(x),breaks=mybreaks,col=mycolors,axes=FALSE);
  box();
}

# functions below are used by show and showmap

acrosspc <- function(x,pos) {
  mn = dim(x);
  m = mn[1];
  mx = (m-2)/5;
  tot = 0*(1:mx);
  aa = 0*(1:mx);
  cc = 0*(1:mx);
  gg = 0*(1:mx);
```

```
  tt = 0*(1:mx);
  pp = which(x[1,] == pos);
  for (i in 1:mx) {
    j = (5*i) - 2;
    tot[i] = x[j,pp];
    aa[i] = x[j+1,pp];
    cc[i] = x[j+2,pp];
    gg[i] = x[j+3,pp];
    tt[i] = x[j+4,pp];
  }
  kp = !is.na(tot);

return(list(tot=tot[kp],aa=aa[kp],cc=cc[kp],gg=gg[kp],tt=tt[kp]));
}

getcoords <- function(x) {
  mn = dim(x);
  m = (mn[1]-2)/5;
  slice = 1:m;
  piece = 1:m;
  for (i in 1:m) {
    j = (5*i) - 2;
    slice[i] = x[j,1];
    piece[i] = x[j,2];
  }
  kpind = !is.na(slice);
  return(list(slice=slice[kpind],piece=piece[kpind]));
}

getxy <- function(piece) {
  x = ceiling(piece/8);
  y = 8*((piece/8) - floor(piece/8));
  if (y == 0) { y = 8; }
  return(list(x=x,y=y));
}

# END SCODE
```

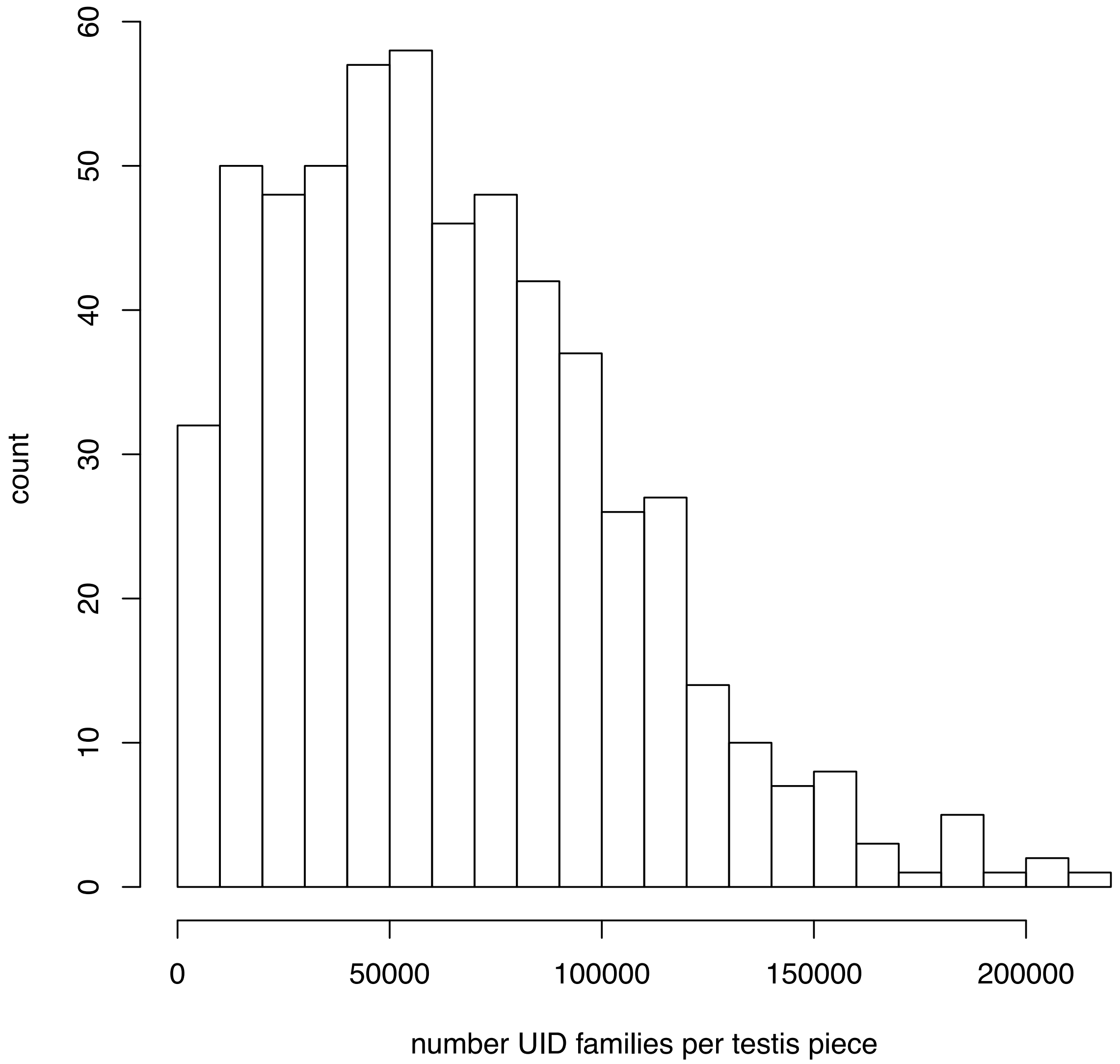

Supp. Figure S1 Histogram of the number of UID families per testis piece. Each UID family represents one strand of a distinct, original (pre-PCR) DNA molecule. The average number of UID families per testis piece is 64 thousand (58,000 is the median). All but 7 of the testis pieces had more than 1,000 UID families, 94% of the testis pieces had more than 10,000 UID families, and 18% had more than 100,000 UID families. 216 thousand was the maximum number of UID families in a single UID piece. In order to minimize the importance of pieces with a small number of UID families, we designated those pieces with fewer than 1,000 UID families as missing data. Three of the 32 pieces from slice two (65 year old donor): #24, #25, #26 and similarly four pieces from the 68 year old donor: one each in slice 3, #32; slice 4, #12; slice 5, #30 and slice 6 #2 provided either no data or fewer than 1,000 UID families and are so marked by the lightest color in the manuscript Figures 1 and H,   and Supp. Figure S5. The number of UID families in each particular testis piece is in Supp_Mat Data 1.

	Eboreime et al., 2016	21 year old in this paper
A>C/T>G	4.4x10-7	3.2x10-7
A>G/T>C	3.3x10-6	4.4x10-6
A>T/T>A	1.4x10-6	1.6x10-6
C>A/G>T	2.2x10-5	1.7x10-5
C>G/G>C	1.1x10-6	1.2x10-6
C>T/G>A (non-CpG)	1.7x10-5	2.2x10-5
C>T/G>A (CpG)	7.4x10-5	no CpGs found in E3

Supp. Figure S2 Background assay rate by mutation type. Previously we published the mutation background of our SSS assay by mutation type (Eboreime et al., 2016). For the data in this paper we computed the background of the assay by only considering data from the youngest (21 year old) testis. This testis had no mutation clusters. We see both the rates and the dependency on mutation type are similar in the two data sets.

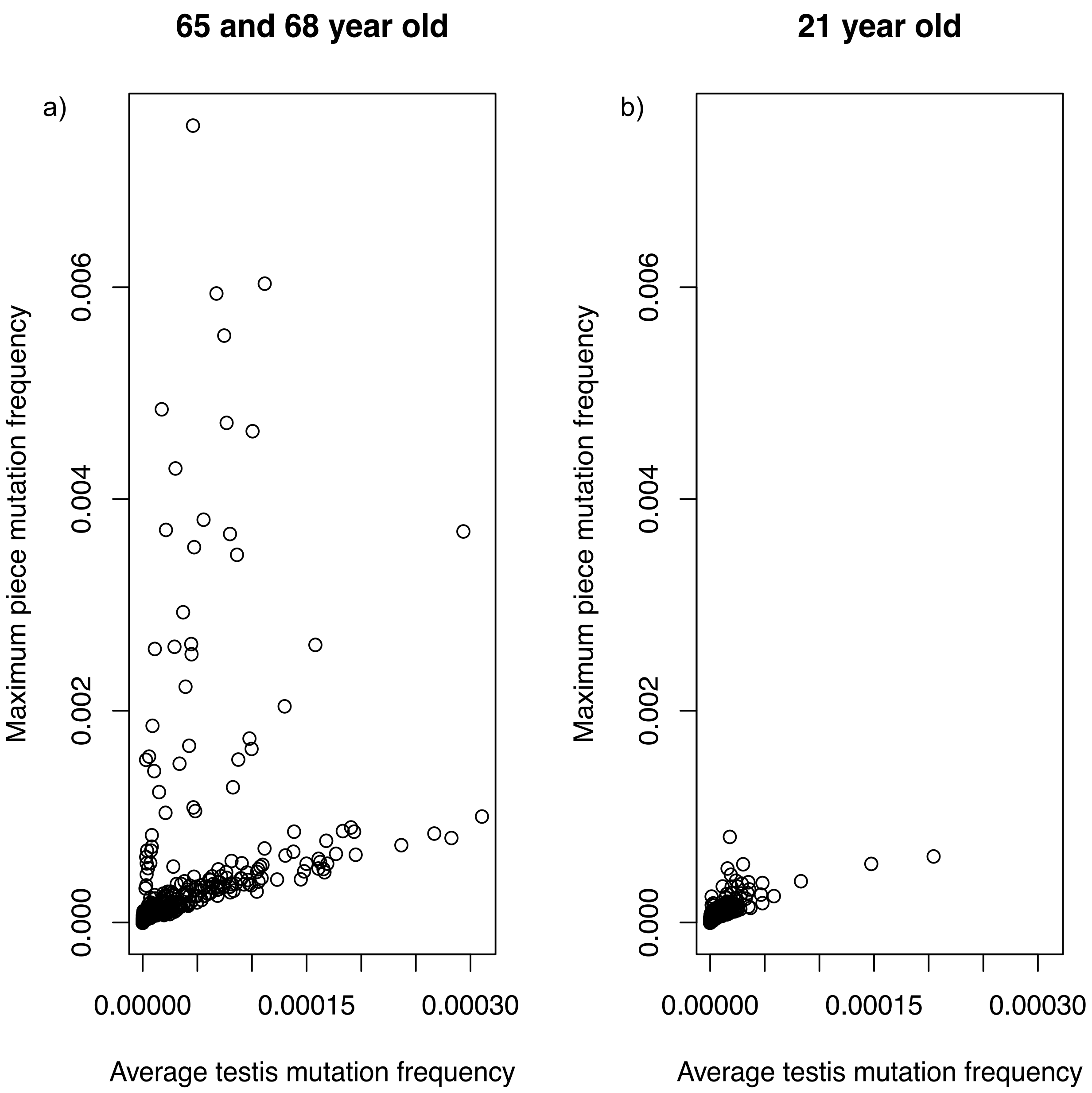

Supp. Figure S3 Maximum testis piece frequency v. average testis frequency separated by age. Qualitatively, the 65 and 68 year old testes (762 data points) have many more variants with relatively high maximum piece frequencies (MaxPF) compared to the 21 year old testis (381 data points).

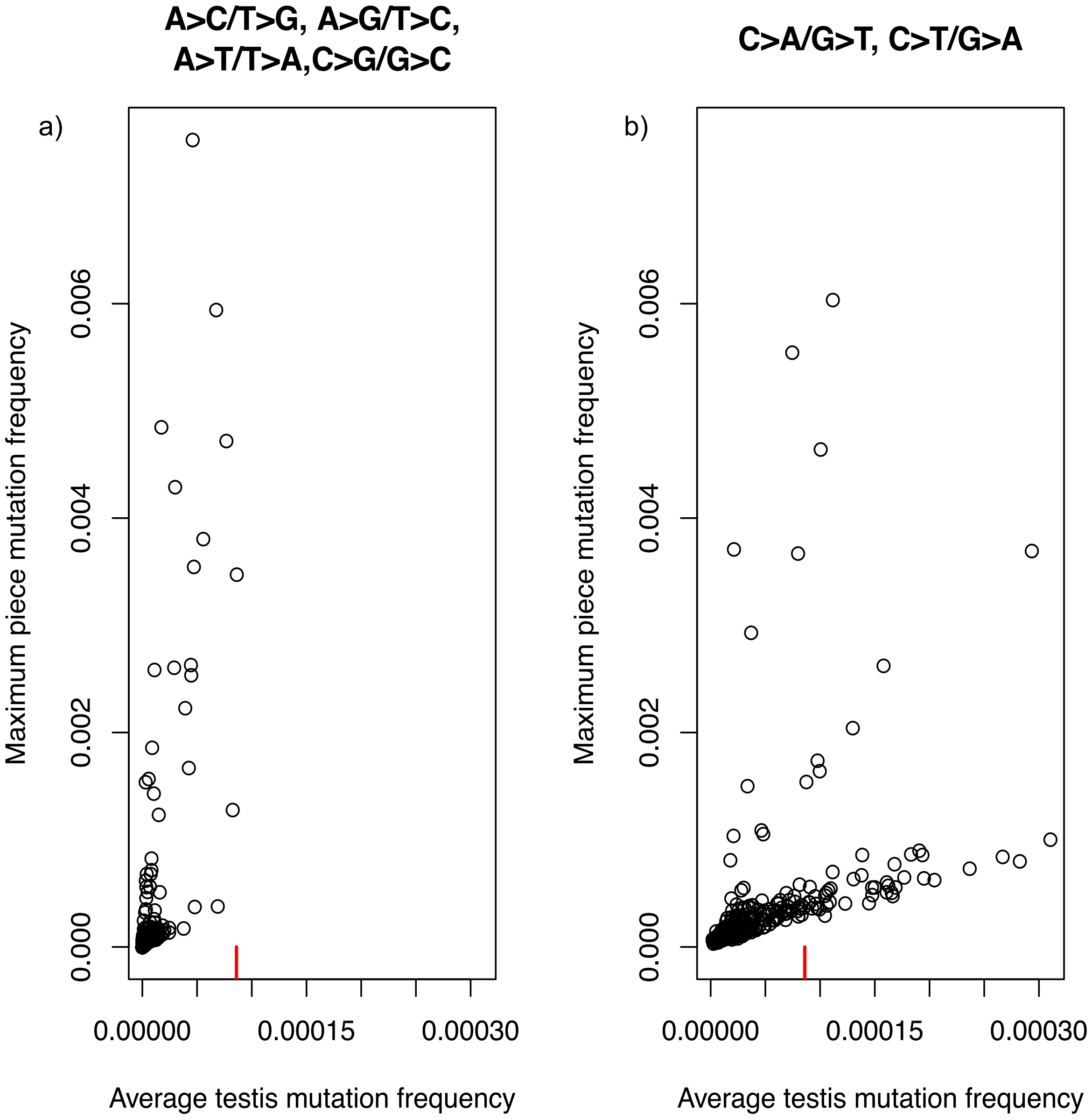

Supp. Figure S4 MaxPF v. average testis frequency separated by variant type. The left-hand plot shows A>C/T>G, A>G/T>C, A>T/T>A, C>G/G>C variants, and the right-hand plot shows C>A/G>T, C>T/G>A variants. The left plot has 825 data points (275 variants x 3 testes), and the right plot has 318 (106 variants x 3 testes). The maximum average testis frequency in the left-hand plot is 8.6×10^{-5} (red notch at the bottom of the figures), while 15% of the variants in right-hand plot have average testis frequencies greater than 8.6×10^{-5}. This difference is due to the higher background of the SSS assay for the C>T/G>A and G>T/C>A variant types (Refs. 1 and 2).

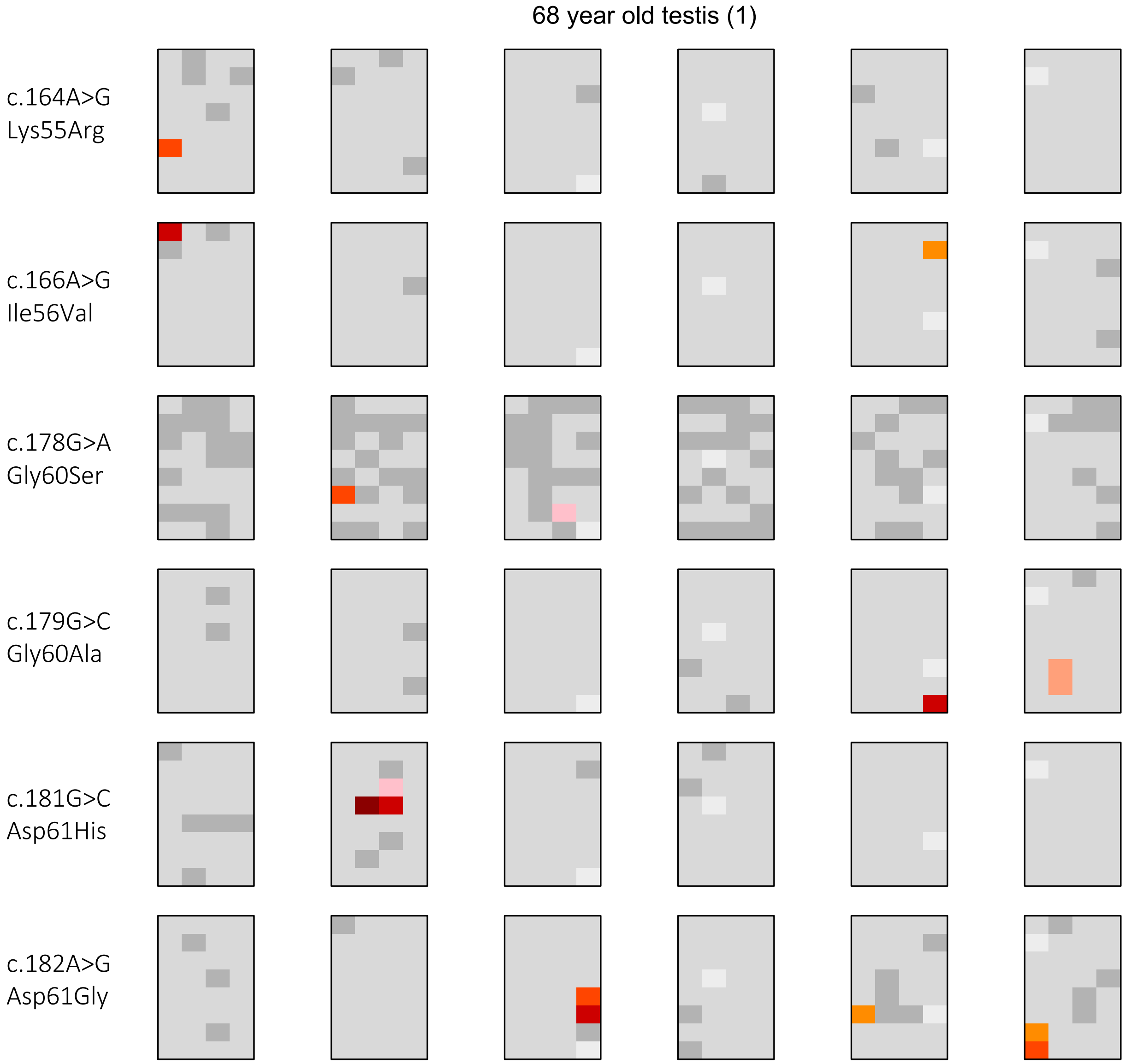
68 year old testis (1)
c.164A>G
Lys55Arg
c.166A>G
Ile56Val
c.178G>A
Gly60Ser
c.179G>C
Gly60Ala
c.181G>C
Asp61His
c.182A>G
Asp61Gly

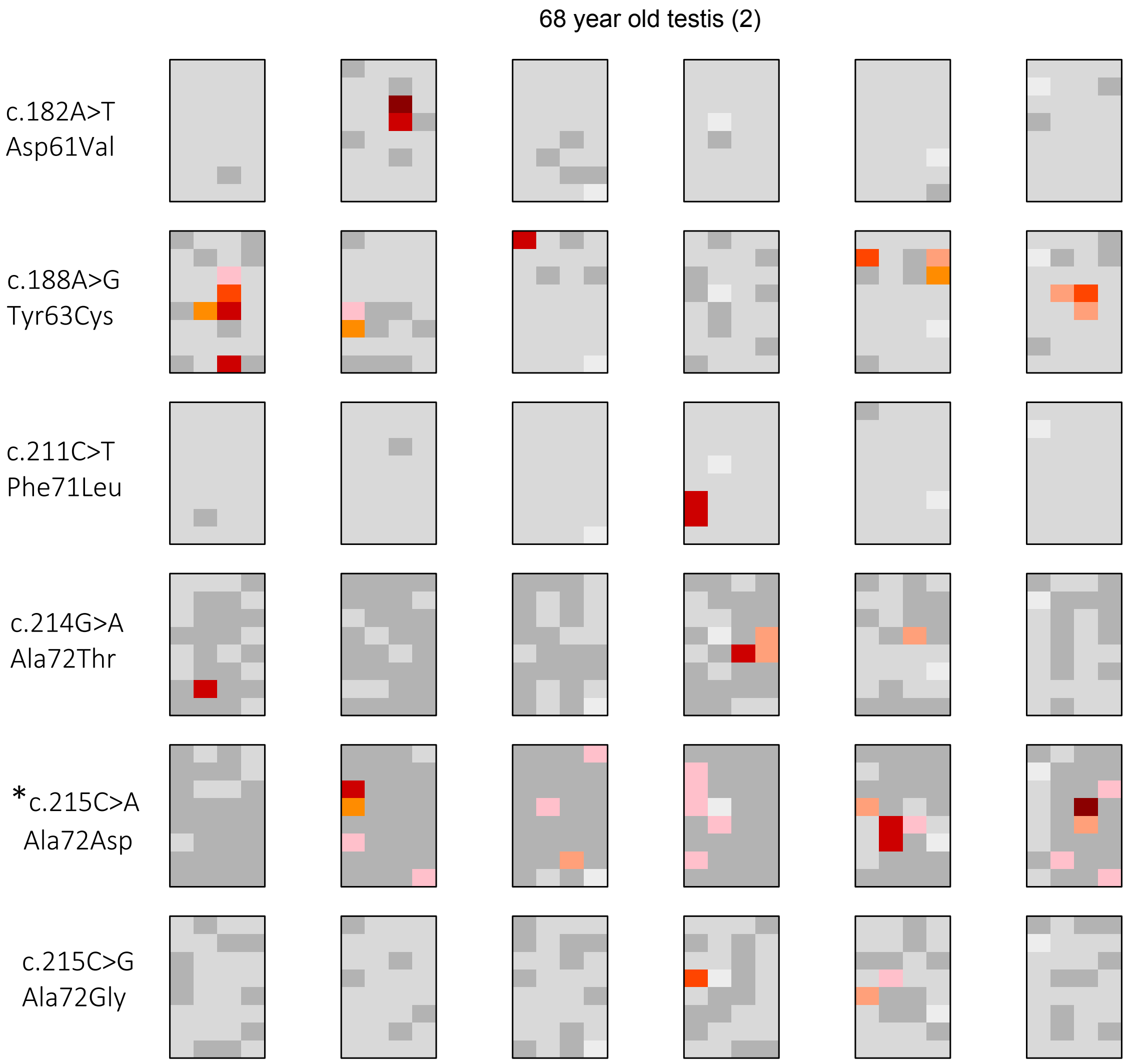
68 year old testis (2)
c.182A>T
Asp61Val
c.188A>G
Tyr63Cys
c.211C>T
Phe71Leu
c.214G>A
Ala72Thr
*c.215C>A
Ala72Asp
c.215C>G
Ala72Gly

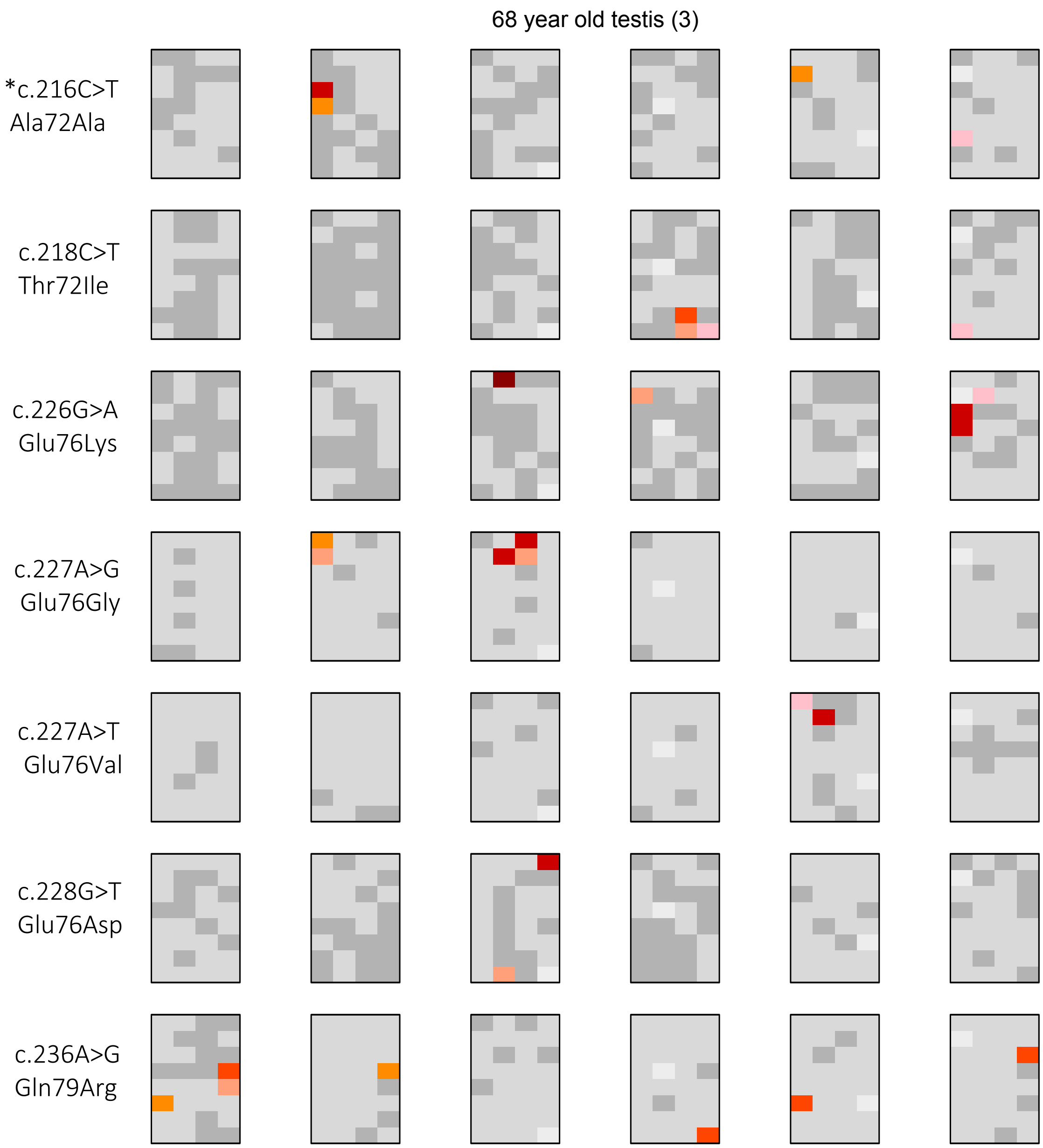
68 year old testis (3)
*c.216C>T
Ala72Ala
c.218C>T
Thr72Ile
c.226G>A
Glu76Lys
c.227A>G
Glu76Gly
c.227A>T
Glu76Val
c.228G>T
Glu76Asp
c.236A>G
Gln79Arg

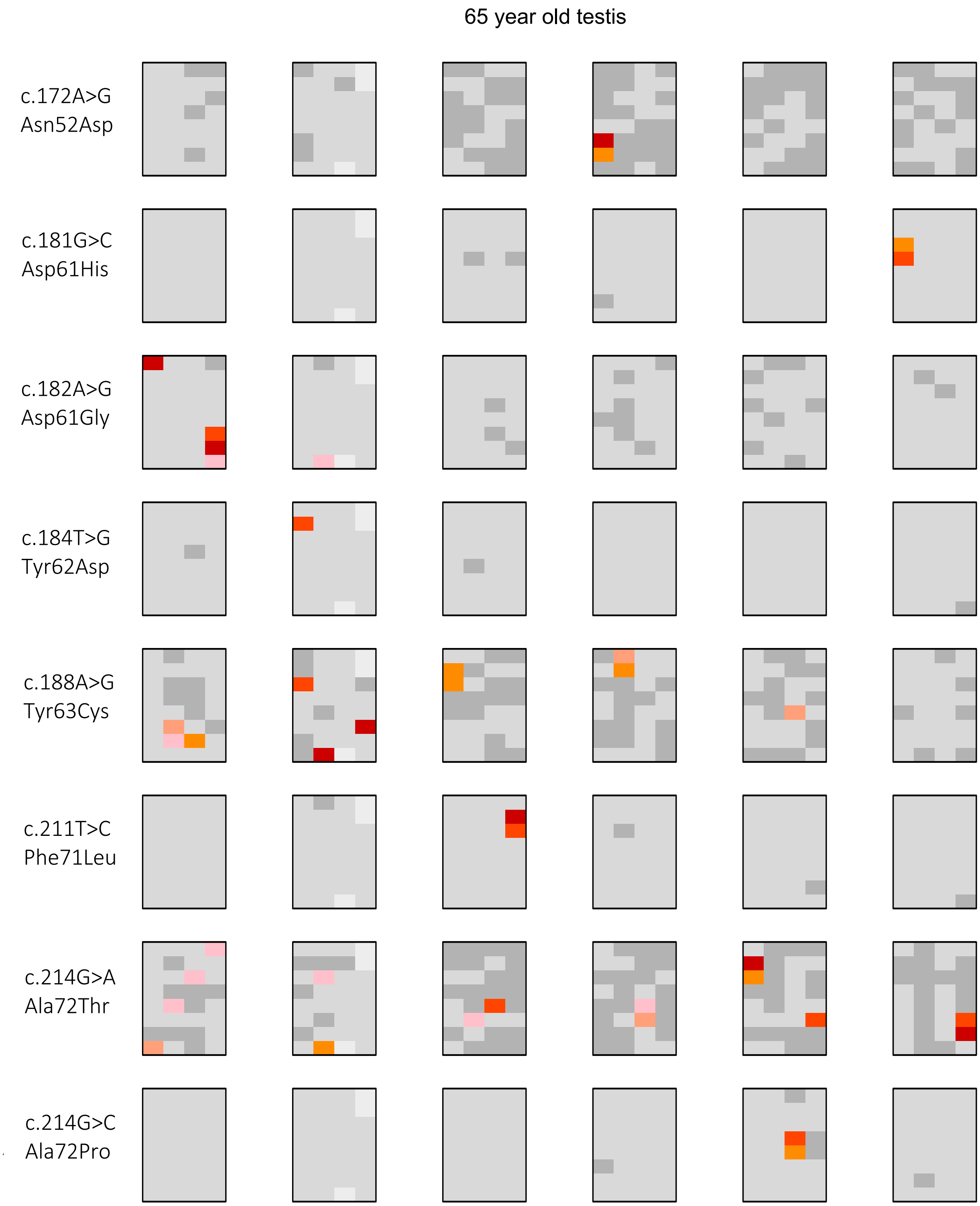
65 year old testis
c.172A>G
Asn52Asp
c.181G>C
Asp61His
c.182A>G
Asp61Gly
c.184T>G
Tyr62Asp
c.188A>G
Tyr63Cys
c.211T>C
Phe71Leu
c.214G>A
Ala72Thr
c.214G>C
Ala72Pro

Supp. Figure S5 Spatial distribution for all significant clusters in the 65 and 68 year old testes. For each testis, the figures are ordered by variant genome position. This figure has four pages: three for the 68 year old and one for the 65 year old. Vertical rectangles show testis slices 1-6 (left to right). The 68 year old has four pieces with missing data (whiteish colored squares) and 65 year old has three pieces.

Note: in the 68 year old testis, the synonymous c.216C>T p.(Ala72Ala) variant hitchhikes with the adjacent non-synonymous sporadic-only cancer variant c. 215C>A p.(Ala72Asp). Reads with the c.216C>T variant in the red and orange colored pieces in the left-most column of slice 2 also have the c.215C>A variant). These two variants are denoted with an *.

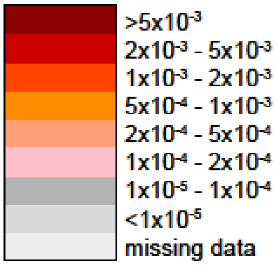

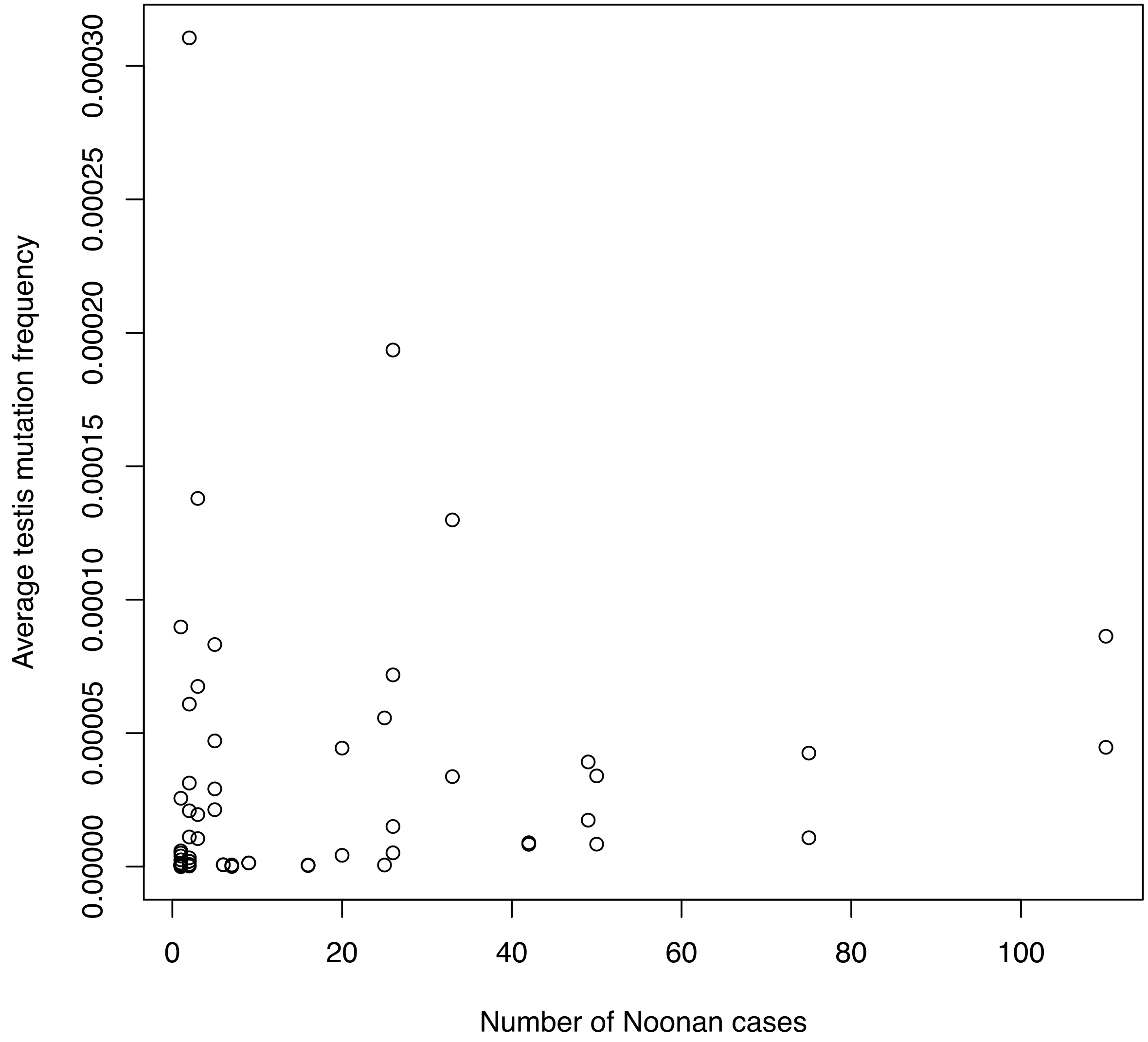

Supp. Figure S6 E3 Noonan case numbers v. average testis frequencies. Considering the 11 recurrent NS1 variant sites and the 19 rarely reported NS1 variant sites in the two older testes (60 total data points), there is no significant correlation between the number of E3 Noonan cases and the average E3 testis variant frequency (p-value 0.38, correlation coefficient 0.12).

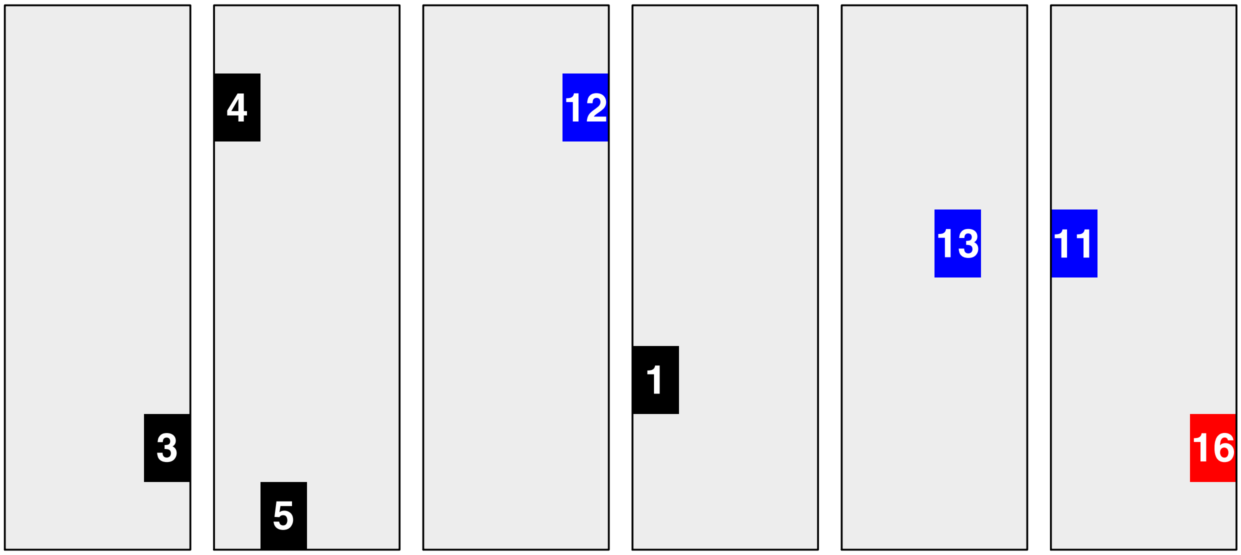

Supp. Figure S7 All 8 significant clusters in the same 65 year old testis. For each variant with a significant cluster, we have indicated MaxFP for that variant. The numbers in the testis pieces correspond to the variant labels in Table 1 of the manuscript. Recurrent NS1 variants are colored black, rarely-recurrent NS1 variants blue, and sporadic-only cancer variants red.

Subject Name	Gene Hit	Subj. Start	Subj. Finish	Query Start	Query End	Length	Score	E-val	%ID
ENSNLEP00000000099 Gibbon (Nomascus leucogenys)	PTPN11	3	590	6	597	592	1172	0	99.32
ENSHGLP00000022310 Naked mole-rat (Heterocephalus glaber)	PTPN11	1	593	1	597	597	1181	0	98.99
ENSCAFP00000013074 Dog (Canis lupus familiaris)	PTPN11	1	593	1	597	597	1179	0	98.66
ENSLAFP00000001842 Elephant (Loxodonta africana)	PTPN11	5	597	5	597	593	1182	0	99.16
ENSECAP00000022175 Horse (Equus caballus)	PTPN11	4	592	5	597	593	1174	0	98.99
ENSOCUP00000003911 Rabbit (Oryctolagus cuniculus)	PTPN11	1	593	1	597	597	1175	0	98.32
ENSMODP00000003804 Opossum (Monodelphis domestica)	PTPN11	1	593	1	597	597	1170	0	97.65
ENSXETP00000009372 Xenopus (Xenopus tropicalis)	PTPN11	5	597	5	597	598	989	0	83.95
ENSPSIP00000003708 Chinese softshell turtle (Pelodiscus sinensis)	ENSPSIG00000003520	1	591	6	597	596	1135	0	94.8
ENSTGUP00000007022 Zebra Finch (Taeniopygia guttata)	ENSTGUG00000006795	1	592	2	597	596	1162	0	97.48

ENSP00000340944 Zebrafish (Danio rerio)	ptpn11a	1	593	1	594	594	1032	0	91.58
ENSAOCP00000008160 Clown anemonefish (Amphiprion ocellaris)	ptpn11a	1	602	1	597	606	1088	0	89.44
ENSAMXP00000049680 Cave fish (Astyanax_mexicanus-2.0)	ptpn11a	1	594	1	597	598	1097	0	91.97
ENSLBEP00000018954 Ballan wrasse (Labrus bergylta)	ptpn11a	1	602	1	597	606	1085	0	89.44
ENSEBUP00000027733 Hagfish (Eptatretus burgeri)	ptpn11a	1	589	1	594	594	877	0	72.9

Supp. Figure S8 Results of amino acid sequence alignments between the subject (Subj.) protein sequence (human SHP-2) and 15 different vertebrate SHP-2 query sequences. Access to the technical details of the alignment BLAST Score, the E-val (probability alignment due to chance) and %ID (per-cent identity between the two proteins) can be found at Http://ensembl.org/index.html

BLAST/BLAT type BLASTP
Query location ZEBRAFISH 1 to 594 (+)
Database location ENSP00000340944 1 to 593 (+)
Genomic location 12 112419112 to 112504761 (+)
Alignment score 1032
E-value 0.0
Alignment length 594
Percentage identity 91.58

Human SH2 1 domain 6-102
Human Exon 3 46-82 K is position of c.164A>G p.(Lys55Arg)

```
H  1 MTSRRWFHPNITGVEAENLLLTRGVDGSFLARPSKSNPGDFTLSVRRNGAVTHIKIQNTG     60
   1 ||||||||||||||||||||||||||||||||||||||||||||||||||||||||||||     60
Z  1 MTSRRWFHPNITGVEAENLLLTRGVDGSFLARPSKSNPGDFTLSVRRNGAVTHIKIQNTG     60

  61 DYYDLYGGEKFATLAELVQYYMEHHGQLKEKNGDVIELKYPLNCADPTSERWFHGHLSGR    120
  61 |||||||||||||||||||||||||||||||||||||||||||||||||||||||||||     120
  61 DYYDLYGGEKFATLAELVQYYMEHHGQLKEKNGDVIELKYPLNCADPTSERWFHGHLSGK    120

 121 EAEKLLTEKGKNGSFLVRESQSHPGDFVLSVRTGDDKTDTSDGKPKVTHVMIRCQHDLKY    180
 121 ||||||||||| |||||||||||||||||||||||||    ||| ||||||||||  |||    180
 121 EAEKLLTEKGKHGSFLVRESQSHPGDFVLSVRTGDDKGESNDGKSKVTHVMIRCQ-ELKY    180

 181 DVGGGEKFDSLTDLVEHYKKNPMVETLGTVLQLKQPLNTTRINAAEIESRVRELSKLAEA    240
 181 |||||| ||||||||||||||||||||||||||||||||||||||||||||||||||||     240
 181 DVGGGERFDSLTDLVEHYKKNPMVETLGTVLQLKQPLNTTRINAAEIESRVRELSKLAET    240

 241 TDKVKQGFWEEFETLQQQECKLLYSRKEGQRPENKNKNRYKNILPFDHTRVVLTDGDVNE    300
 241 ||||||||||||||||||||||||||||||| ||||||||||||||||||||| ||| ||    300
 241 TDKVKQGFWEEFETLQQQECKLLYSRKEGQRQENKNKNRYKNILPFDHTRVVLHDGDPNE    300

 301 QGSDYINANLIMPDNEAKSNNSKLKRSYIATQGCLQNTISDFWRMVFQENSRVIVMTTKE    360
 301   ||||||| |||  | | |||| | ||||||||||||  ||||||||||||||||||||    360
 301 PVSDYINANIIMPEFETKCNNSKPKKSYIATQGCLQNTVNDFWRMVFQENSRVIVMTTKE    360

 361 VERGKSKCVKYWPDVSALKEYGAMRVRNVKETMAHDYILRELKLSKVGQGNTERTVWQYH    420
 361 ||||||||||||||  |||||| ||||||||  |||| ||||||||||||||||||||||    420
 361 VERGKSKCVKYWPDEYALKEYGVMRVRNVKESAAHDYTLRELKLSKVGQGNTERTVWQYH    420

 421 FRAWPDHGVPGDPGGVLDFLEEVKLKQEGITGAGPIVVHCSAGIGRTGTFIVIDILIDII    480
 421 || ||||||| ||||||||||||  ||| |  ||| ||||||||||||||||||||||||    480
 421 FRTWPDHGVPSDPGGVLDFLEEVHHKQESIMDAGPVVVHCSAGIGRTGTFIVIDILIDII    480

 481 REKGVDCDIDVPKTIQMVRSQRSGMVQTEAQYRFIYMAVQHYIETLQRRIEEEQKSKIKG    540
 481 ||||||||||||||||||||||||||||||||||||||||||||||||||||||||| ||    540
 481 REKGVDCDIDVPKTIQMVRSQRSGMVQTEAQYRFIYMAVQHYIETLQRRIEEEQKSKRKG    540

 541 REYTNIKYSLSDLSGGDQSPLPPCTPTPTCADMRDDSSRVYENVGLMQQQKSHR          594
 541  ||||||||| |   ||||||||||||| || || || |||||||||||||| |          594
 541 HEYTNIKYSLADQTSGDQSPLPPCTPTPPCAEMREDSARVYENVGLMQQQKSFR          594
```

Supp. Figure S9 Alignment of human and zebrafish SHP-2 proteins. See https://ensembl.org/index.html for additional details

	Transitions	Transversions
E3	12	18
Rest of *PTPN11*	41	35

Supp. Figure S10 Number of NS1 variants in E3 and the rest of *PTPN11* (not including E3) that are transitions and transversions. There is not sufficient evidence to reject the null hypothesis that the transition/transversion ratio for NS1 variants is the same in the two genomic regions (chi-squared test, p-value 0.20).

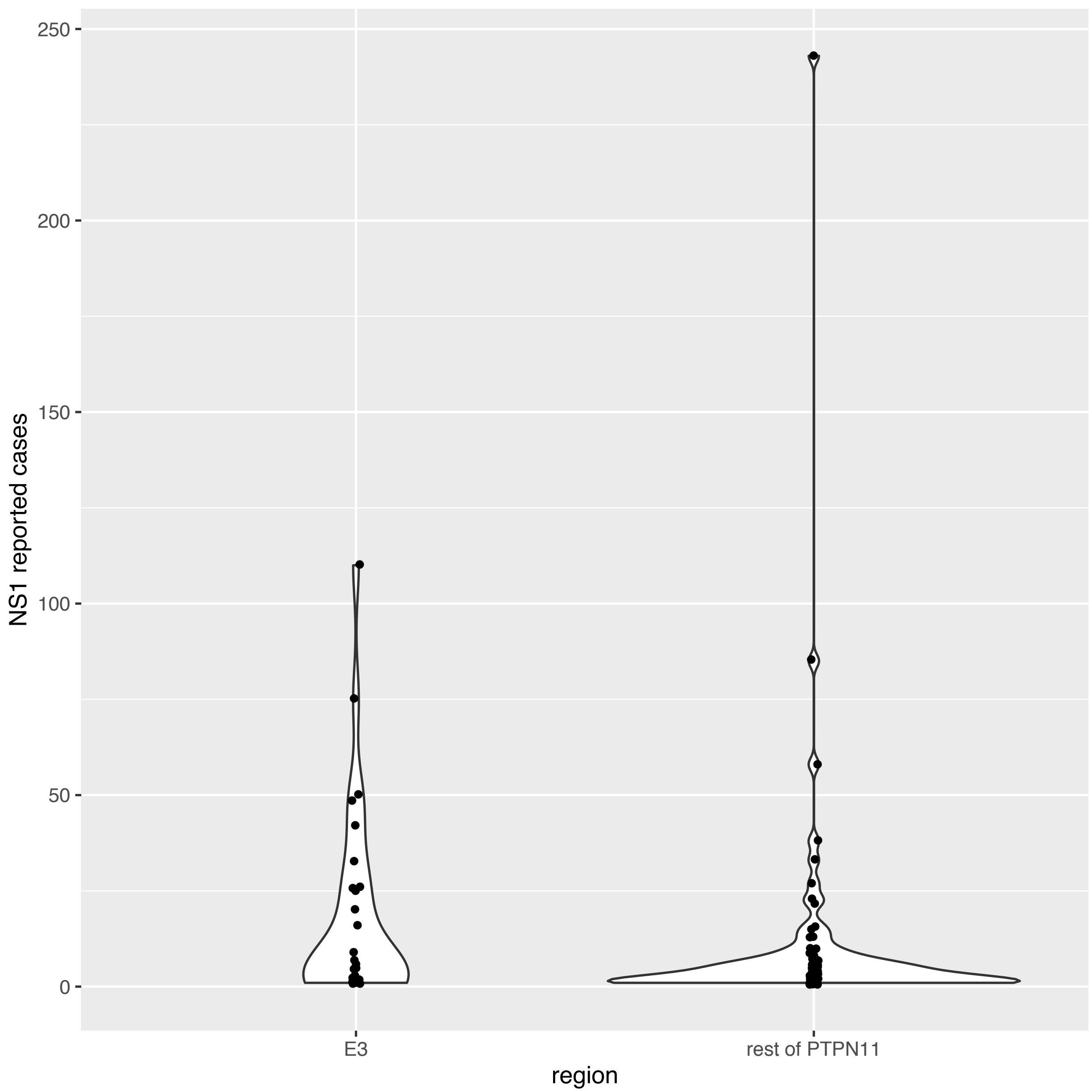

Supp. Figure S11 Number of reported NS1 cases for each of the variants in E3 and the rest of *PTPN11*. There is not sufficient evidence to reject the null hypothesis that the distribution of case numbers is the same in the two genomic regions (Wilcoxon-Mann-Whitney test, p-value 0.06). We used the Wilcoxon-Mann-Whitney test (which only uses ranks) because the reporting of NS1 cases is possibly biased (researchers may be more likely to report newly discovered NS1 variants than well-known variants in NSEuronet).

Data Source	Substrate	Protein variant	Raw activity	Relative activity	NS1 cases	Cancer cases	Clusters
(Keilhack et al., 2005)	RCML	WT	0.17	1			
		Thr42Ala	0.48	2.82	27	2	
		Asp61Gly	1.63	9.59	49	6	*(65,68)
		Thr73Ile	1.25	7.35	33	20	*(68)
		Glu76Asp	0.42	2.47	21	0	
		Asp106Ala	0.21	1.24	23	0	
		Glu139Asp	0.33	1.94	38	5	
		Asn308Asp	0.5	2.94	241	3	
		Asn308Ser	0.21	1.24	85	2	
		Gln506Pro	0.19	1.12	7	2	
		Asp61Tyr	3	17.65	0	54	
		Glu76Lys	4.38	25.76	0	115	*(68)
	RCML+IRS-1	WT	0.44	1			
		Thr42Ala	2.88	6.55	27	2	
		Asp61Gly	2.88	6.55	49	6	*(65,68)
		Thr73Ile	1.79	4.07	33	20	*(68)
		Glu76Asp	0.35	0.8	21	0	
		Asp106Ala	1.02	2.32	23	0	
		Glu139Asp	2.25	5.11	38	5	
		Asn308Asp	0.83	1.89	241	3	
		Asn308Ser	0.4	0.91	85	2	
		Gln506Pro	0.27	0.61	7	2	
		Asp61Tyr	2.92	6.64	0	54	
		Glu76Lys	3.81	8.66	0	115	*(68)
(Tartaglia et al., 2006)	pNPP	WT	0.33	1			
		Thr42Ala	0.38	1.15	27	2	
		Ala72Ser	0.67	2.03	26	2	
		Thr73Ile	0.88	2.67	33	20	*(68)
		Glu76Asp	1.04	3.15	21	0	
		Glu139Asp	0.42	1.27	38	5	
		Ile282Val	0.83	2.52	22	0	
		Asn308Asp	0.46	1.39	241	3	
		Met504Val	0.21	0.64	60	1	
		Ala72Val	1.54	4.67	0	79	
		Glu76Lys	2.64	8	0	115	*(68)
	pNPP+BTAM	WT	0.63	1			
		Thr42Ala	1.19	1.89	27	2	
		Ala72Ser	1.84	2.92	26	2	
		Thr73Ile	3.13	4.97	33	20	*(68)
		Glu76Asp	2.08	3.3	21	0	
		Glu139Asp	2.21	3.51	38	5	
		IIle282Val	1.42	2.25	22	0	
		Asn308Asp	1.29	2.05	241	3	
		Met504Val	1.44	2.29	60	1	
		Ala72Val	2.88	4.57	0	79	
		Glu76Lys	3.04	4.83	0	115	*(68)

Supp. Figure S12 Correlation of SHP-2 activity and NS1 recurrence. The Relative activity data are plotted in Figure p of   the manuscript (also see Methods in manuscript).

For library construction each of the 10 SSS reactions (per testis piece) contained $1x10^5$ genomes of purified DNA, 1x GC Phusion Buffer (1.5mM $MgCl_2$), 300nM of each of the first round primers (see below), 80µM dNTP, Phusion Hot Start High Fidelity DNA Polymerase (0.02U/ul), and 0.26x SYBR green, 2.6µM ROX solution in a 45 µL reaction. After a 98°C 1 min initial denaturation, two PCR cycles were run (98°C 30sec, 62°C 4min, 72°C 1min) followed by 72°C for 5 minutes in a MJ Research Opticon 2 QPCR instrument. These 1st round PCR primers were removed by adding Agencourt AMPure XP paramagnetic beads in a buffer optimized to selectively bind DNA fragments 100 bp and larger. After 5 minutes at room temperature, the plate was placed on a magnet for 2 minutes, washed twice with 70% ethanol, and allowed to dry for 9-10 minutes in a biosafety cabinet.

The DNA on the dried magnetic beads was resuspended in a second round PCR buffer which was identical to the first round buffer except the first round primers were replaced by 550nM of each second round primer also in a 45 µL reaction. Twenty-eight additional cycles of PCR were performed (98°C 1 min initial denaturation, 98°C 10sec, 72°C 15 sec ending with 72°C for 5 min). Both second round primers (see Supp_Mat Methods 2) contained sequences complementary to the 5' most portion of the first round PCR product at the 3' end. The 5' end of the second round primers carried the sequences needed for binding to the Illumina flow cell. Following the second round, 0.8x PEG 8000 (20%) dissolved in 2.5M NaCl (required for a bead DNA binding step) was added to each reaction, allowed to sit at room temperature for 5 minutes, exposed to the magnet for 2 minutes, washed twice with 70% ethanol, and allowed to dry for 5-6 minutes. The DNA was eluted in 10µL of water and the expected 317bp final product from each of the ten reactions for each piece were pooled. For the final library, all 192 pooled PCR products for the testis were combined in equal amounts based on Nanodrop analysis. The pooled and concentrated (SpeedVac) sample was evaluated (Bioanalyzer 2100, Agilent Technologies) to quantify the final amount of DNA in the library. The DNA was then loaded on a NextSeq 500 and 150bp paired-end sequencing was performed

Supp_Mat Methods 1 Technical aspects of SSS library construction.

Primers for first two PCR cycles

Example of a "one-sided" barcode where the barcode is just on the reverse primer. For all three testes, one-sided barcodes were used for slices 2 – 6. The "ACGCTTGA" barcode is used for slice 2, testis piece 1 (see Supp_Mat Data1 for the complete list of barcodes used).

SSS Noonan E3 Reverse Primer:
5'CGGCATTCCTGCTGAACCGCTCTTCCGATCTACGCTTGANNNNNNTCTTTTAATTGCCCGTGATGTTCC

SSS Noonan E3 Forward Primer:
5'CGACGCTCTTCCGATCTNNNNNNNNNNNNNNTATTTGTCCCCTTGCCTCCCT

Example of a "split" barcode where the barcode is split between the two primers. For all three testes, split barcodes were used for slices 1. The "CAGACTAT" barcode (the first four bases are on the forward primer and the next four bases are on the reverse primer) is used for slice 1, testis piece 1.

SSS Noonan E3 Reverse Primer:
5'CGGCATTCCTGCTGAACCGCTCTTCCGATCTCTATNNNNNNNNNNTCTTTTAATTGCCCGTGATGTTCC

SSS Noonan E3 Forward Primer:
5'CGACGCTCTTCCGATCTCAGANNNNNNNNNNTATTTGTCCCCTTGCCTCCCT

Color key:
PORTION OF ILLUMINA SEQUENCING REGION
BARCODE
UNIVERSAL IDENTIFIER
PTPN11-SPECIFC TARGETING SEQUENCES

Second Round Primers for remaining PCR cycles

Second Round Forward Primer:
5'AATGATACGGCGACCACCGAGATCTACACTCTTTCCCTACACGACGCTCTTCCGAT*C*T

Second Round Reverse Primer:
5'CAAGCAGAAGACGGCATACGAGATCGGTCTCGGCATTCCTGCTGAACCGCTCTTCCG*A*T

Color key:
FLOWCELL ADAPTER SEQUENCES
OVERLAPPING PORTION OF ILLUMINA SEQUENCING REGION
(* phosphorothioate linkage)

Illumina sequence reads

The two reads overlap for 100 base pairs.

Example of a “one-sided” barcode. For this example, the barcode is “ACGCTTGA” on R2 as in the one-sided example above.

```
R1 5’ NNNNNNNNNNNNNNTATTTGTCCCCTTGCCTCCCTTTCCAATGGACTATT
      TTAGAAGAAATGGAGCTGTCACCCACATCAAGATTCAGAACACTGGTGAT
      TACTATGACCTGTATGGAGGGGAGAAATTTGCCACTTTGGCTGAGTTGGT

R2 5’ ACGCTTGANNNNNNTCTTTTAATTGCCCGTGATGTTCCATGTAATACTGG
      ACCAACTCAGCCAAAGTGGCAAATTTCTCCCCTCCATACAGGTCATAGTA
      ATCACCAGTGTTCTGAATCTTGATGTGGGTGACAGCTCCATTTCTTCTAA
```

Example of a “split” barcode. For this example, the barcode is “CAGACTAT” (first four bases on R1, second four bases on R2) as in the split example above.

```
R1 5’ CAGANNNNNNNNNNTATTTGTCCCCTTGCCTCCCTTTCCAATGGACTATT
      TTAGAAGAAATGGAGCTGTCACCCACATCAAGATTCAGAACACTGGTGAT
      TACTATGACCTGTATGGAGGGGAGAAATTTGCCACTTTGGCTGAGTTGGT

R2 5’ CTATNNNNNNNNNNTCTTTTAATTGCCCGTGATGTTCCATGTAATACTGG
      ACCAACTCAGCCAAAGTGGCAAATTTCTCCCCTCCATACAGGTCATAGTA
      ATCACCAGTGTTCTGAATCTTGATGTGGGTGACAGCTCCATTTCTTCTAA
```

Color key:
BARCODE
UNIVERSAL IDENTIFIER
PTPN11-SPECIFC TARGETING SEQUENCES
E3 REFERENCE SEQUENCE (the 100 underlined bases overlap between R1 and R2)

Supp_Mat Methods 2 Examples of E3 PCR primers using the Safe Sequencing System (SSS) method, and the resultant Illumina sequence reads**.** The first two cycles of PCR produce uniquely barcode and UID tagged E3 PCR product from each original template molecule (the barcodes shown in the figure are only two of the many possible barcodes available). The unused first cycle UID primers are removed and two new second round primers are introduced. These new primers are complementary to the 5’ tails of the first round primers so it only amplifies products of the first round which acquired a UID. The result of this second round PCR are many different UID families and each family is originated from one of the original genomic template molecules.

We wrote Perl scripts (available from P.C. on request) to analyze the sequencing data. We only considered reads with fewer than 5% of bases different from the reference sequence, and quality scores of at least 30 at all nucleotide sites in the barcode and UID (Eboreime et al., 2016). We clustered reads with the same UID and barcode into families considering only those families with at least 3 paired reads with the same UID and barcode. At each nucleotide site, the variant frequency is calculated by dividing a numerator by a denominator. The denominator is the number of UID families that, at this particular nucleotide site, have at least 3 (unpaired) reads with quality scores of at least 30 (because of this quality restriction the denominator may be different at different sites). The numerator is the number of UID families that at this particular site have at least 3 (unpaired) reads with quality scores of at least 30 and that 95% of these reads have the same base which is different than the reference (sometimes called a super-mutant (Kinde et al., 2011)).

Note: All of the sequencing reads in this study are paired. For 100 bases in the 127 base pair E3 sequence, these paired reads overlap (see Supp_Mat Methods 2 for an illustration). Therefore the two paired reads combine to sequence 227 E3 nucleotides. The 5% rule allows for 227x.05=11 or fewer bases to be different from the reference sequence. To count a UID family there must be at least 3 paired reads with the same UID and barcode. When looking for super-mutants within a UID family at nucleotide sites where the paired reads overlap, we consider the paired reads separately. For example, consider the case where there are three paired reads with the same UID and barcode. Assume that at a nucleotide site where the paired reads overlap, all six of these (unpaired) reads have quality scores greater than 30. Further assume at this site five of these (unpaired) reads have the same sequence as the reference and one (unpaired) read has a different sequence (so necessarily one set of paired reads disagree at this site). Then in the variant frequency calculation at this site this UID family would contribute one to the denominator (because there are at least three unpaired reads that meet the quality threshold) and zero to the numerator (because one out of six is less than 95%).

Supp_Mat Methods 3 Measuring mutation frequency details

For each testis and every possible variant we calculated two numbers: the average variant frequency across all the testis pieces and the frequency of the testis piece with the maximum frequency (denoted MaxPF). The idea is that we will use the average frequency to fit the one model parameter. We will then simulate the model with this parameter value and compare the simulated MaxPF values to the MaxPF observed in the testis.

For the 21 year old testis, the largest MaxPF for all of the variants is 8.1×10^{-4}. Consequently, we used 10^{-3} as a threshold. For the 65- and 68-year-old testes, there are a total of 37 variants with a MaxPF greater than 10^{-3}. Separately for each of these 37 variants: (1) we computationally estimated the maximum likelihood model parameter that best matches the observed average testis frequency, (2) we simulated the model with this parameter value keeping only those simulations where the simulated average testis frequency was within 5% of the observed average testis frequency (we simulated enough times so that there would be at least 10,000 kept simulations), (3) for these kept simulations we compared the distribution of simulated MaxPFs to the observed MaxPFs, (4) if the observed MaxPF was in the top (1/37%) of this distribution (so the top 1% after the Bonferroni correction for 37 multiple tests) then the neutral model was rejected and the variant was considered significantly clustered. Because the Bonferroni correction is conservative, the figures for some of the variants that are not determined to be significantly clustered (in particular those just below the red line in Figure 0B) are not strictly gray like Figure 1B but have some testis pieces that are more brightly colored.

Supp_Mat Methods 4 Using neutral models to identify significant clusters

The E3 significant testis clusters could not be explained by the neutral model (simulations of this model that match the observed average testis variant frequency had much lower MaxPFs than were observed). Previously (see (Arnheim & Calabrese, 2016)) we have considered other neutral models, but they could not explain the variant clusters either.

In the selection model we fixed the mutation rate per cell division so that for the case when the selection parameter is zero (in this case the model is the same as the neutral symmetric hot spot model), the variant frequency in a 21-year-old testis matches the known genome average bp mutation rate per generation (Rahbari et al., 2016; Segurel et al., 2014). To infer the selection parameter, we simulated the model with different selection parameter values to find those values that best match the observed MaxPFs and observed average testis frequencies for those testes with significant clusters. For the clustered variants, the inferred values of the selection parameter are all near 0.003. The previously studied RAMP/PAE variants at the *FGFR2* (MIM# 176943), *FGFR3* (MIM# 134934) and *RET* (MIM# 164761) receptor tyrosine kinase (RTK) loci (Choi et al., 2008, 2012; Qin et al., 2007; Yoon et al., 2013) included variant clusters in testes with a wider range of donor ages where the inferred selection parameters varied from 0.002 to 0.008. We used the mean of these values (0.005) to calculate NS incidence since the age of fathers in the overall demographic data was better represented by these testes than in the two older testes reported here.

Since there was no evidence for a difference between the E3 variants and the other NS1 variants in terms of variant type (Ti/Tv, Figure Sмл) or between the average testis variant frequency and the number of reported Noonan syndrome cases (Figure Sc), we assumed, for simplicity, the same selection parameter for all 110 reported NS1 variants. Based on Figure п and Figure Sт we also assumed each potential variant cluster arose and evolved independently.

In order to test the extent to which germline selection on NS1 variants throughout *PTPN11* can explain the overall high NS birth rate (1/1,000- 1/2,500) we used the selection model and US demographic data. To do this we simulated the selection model for a distribution of ages based on the demographic data for the age of fathers. We combined the birth rate as a function of the age of the father (Martin et al., 2015) and the male population by age (Census) to determine the distribution of the age of fathers in the US. We randomly selected men's ages from this distribution. For each age we independently simulated the selection model once for each NS1 variant site. The probability a man of this age has an affected child is then the sum of these 110 average testis variant frequencies. We thus predicted the incidence rate for spontaneous NS1 and then, in order to predict the NS birth rate, we multiplied this rate by four since 50%-60% of NS cases arise from *PTPN11* variants (Maheshwari et al., 2002; Tartaglia et al., 2002; Tartaglia et al., 2001) and >50% NS cases are caused by *de novo* variants in the unaffected father's germline(Tartaglia et al., 2004; Tartaglia et al., 2006; Tartaglia et al., 2001).

Supp_Mat Methods 5 Details of Selection model estimate of NS incidence

Using *in silico* methods we examined the possible functional consequences of the previously unreported E3 base substitution (c.164A>G) that would lead to a *presumptive* p.(Lys55Arg) variant. We first used *PolyPhen-2 v2.2.3r406* (http://genetics.bwh.harvard.edu/pph2/dbsearch.shtml). Two different versions were used for evaluation. With HumDiv (preferable for evaluating rare alleles, analysis of natural selection and alleles found in dense GWAS regions) p.(Lys55Arg) was predicted to be “benign” with a score of 0.283 (sensitivity: 0.91; specificity: 0.89). Using HumVar (preferred for diagnostics of Mendelian diseases by distinguishing variants with drastic effects from all of the remaining human variation) a “benign” prediction was made (score of 0.366 (sensitivity: 0.85; specificity: 0.78).

The evaluation of p.(Lys55Arg) with Provean (http://provean.jcvi.org/index.php) scored -2.344 (which is above the -2.5 threshold) thereby predicting it was “neutral”. Note: the web-based version of Provean has been retired but can be downloaded (https://www.jcvi.org/research/provean).

Next we used PON-P2 (http://structure.bmc.lu.se/PON-P2/) with the default settings. The *presumptive* p.(Lys55Arg) variant was found to have a “Probability Of Pathogenicity” score of 0.779 with a SE of 0.097; the clinical prediction was given as “unknown”.

Finally, we used the e!Ensembl BLAST/BLAT alignment tool (http://ensembl.org/) with the default settings and examined the conservation of the p.(Lys55) amino acid over a wide range of vertebrate animals over 400 million years of evolution. Pairwise alignment was carried out between the amino acid sequence of human SHP-2 (UniProtKB-(Q06124) and the SHP-2 homolog of each of 15 query species. The Data are shown in Supp. Figure y and Supp. Figure ɸ and are discussed in the manuscript.

We suggest that the “benign” and “neutral” predictions came about because of how PolyPhen-2 and Provean evaluated the evolutionary conservation of the p.(Lys55) amino acid. PolyPhen-2 searches for homologs in different species using a 71 amino acid region with the query amino acid in the middle. Pon-P2 searches for homologs with the complete protein sequence. In the case of *PTPN11* p.(Lys55) this difference is critical. Lys 55 is located in the the highly conserved N-SH2 domain which is found in many other phosphatases that are **not** related to SHP-2. The final PolyPhen-2 or Provean aligments therefore include many phosphatases from species that carry an N-SH2 domain but are not in the SHP-2 family thereby often showing variants at codon 55. Our e!Ensembl BLAST/BLAT alignment search with human SHP-2 showed no variants of p.(Lys55Arg) over 400 million years of evolution and supports the PON-P2 conclusion that there is a high probability of pathogenicity.

Supp_Mat Methods 6 *In silico* analysis of *presumptive* variant p.(Lys55Arg)

In Figure p, we did a Wilcox -Mann-Whitney test to examine the null hypothesis that the normalized relative phosphatase activity was no different for the NS1 variants and the cancer variants. Since one of these variants (c.218C>T p.(Thr73Ile) is unusual in that it is both a highly recurrent NS1 variant (33 cases) and a frequent contributor to sporadic cancer variants (20 cases). We did the test separately three different ways: categorizing p.(Thr73Ile) only as a cancer variant, only a NS1 variant, and removing it completely. In all three cases the p-value was less than $3x10^{-5}$.

Supp_Mat Methods 7 Statistical test details for constructing Figure p in the manuscript

The mathematical explanation for the data points lying roughly in a straight line in Figure 6 is that most of the RAMP/PAE mutants in a testis are generally found in the piece with the highest MaxPF. Since there are 192 testis pieces, log(ave) ~ log(max/192) = log(max) – log(192) = log(max) – 2.3. So the log average frequency (x-axis) is roughly the log maximum frequency (y-axis) minus 2.3.

If we make the simplifying assumption that the 110 NS1 variants (other than p.Asn308Asp in exon 8) contribute equally then the E3 variants (blue) have apparent substitution rates approximately 25 to 50 times greater than the known genome average. We arrive at this estimate by the following calculation: estimated NS disease incidence (Allanson & Roberts, 2001 Nov 15 [Updated 2016 Feb 25]; Tartaglia et al., 2010; Zenker, 2017) x percentage of sporadic cases (Tartaglia et al., 2004; Tartaglia et al., 2006; Tartaglia et al., 2001) x percentage that are *PTPN11* variants (Maheshwari et al., 2002; Tartaglia et al., 2002; Tartaglia et al., 2001) x percentage not p.Asn308Asp (Tartaglia & Gelb, 2005) (1/110).

Supp_Mat Methods 8 Mathematical details to explain the shape of the data in Figure 6 and the estimate of the apparent NS1 mutation rate relative to the known human rate

Supp_Mat Data1.xlsx Barcodes and number of UID families for each testis piece. For all three testes, for slice 1 the barcodes are split between two primers, and for slices 2 – 6 the barcodes are on just one primer (see Supp_Mat Methods 2 for details). The sequence data for this study has been submitted to the NCBI BioProject database with accession number PRJNA517482. There are five separate sequencing runs: SRR8497672 for testis 60649 (65 y.o.) slices 1 – 2, SRR8497673 for testis 60649 slices 3 – 6, SRR21613746 for testis 63878 (21 y.o.) slices 1 – 4, SRR21613906 for testis 60891 (68 y.o.) slices 3 – 6, and SRR8497671 for testis 60891 slices 1 – 2 and testis 63878 slices 5 – 6. Some barcodes are used multiple times, but never in the same sequencing run.

Supp_Mat Data2.xlsx For each possible E3 variant the number of Noonan and cancer cases and summary of testis data.

Supp_Mat Data3.csv Variant frequencies for all testis pieces. See Note S1 for format explanation.

References for Supporting Information

Allanson, J. E., & Roberts, A. E. (2001 Nov 15 [Updated 2016 Feb 25]). Noonan Syndrome. *GeneReviews™ [Internet]. Seattle (WA): University of Washington, Seattle; 1993-2017*. https://www.ncbi.nlm.nih.gov/books/NBK1124/

Arnheim, N., & Calabrese, P. (2016). Germline Stem Cell Competition, Mutation Hot Spots, Genetic Disorders, and Older Fathers. *Annu Rev Genomics Hum Genet*, *17*, 219-243. https://doi.org/10.1146/annurev-genom-083115-022656

Census, U. Annual Estimates of the Resident Population for Selected Age Groups by Sex for the United States, States, Counties and Puerto Rico Commonwealth and Municipios: April 1, 2010 to July 1, 2017. https://factfinder.census.gov/faces/tableservices/jsf/pages/productview.xhtml?src=bkmk

Choi, S. K., Yoon, S. R., Calabrese, P., & Arnheim, N. (2008). A germ-line-selective advantage rather than an increased mutation rate can explain some unexpectedly common human disease mutations. *Proc Natl Acad Sci U S A*, *105*(29), 10143-10148. http://www.ncbi.nlm.nih.gov/entrez/query.fcgi?cmd=Retrieve&db=PubMed&dopt=Citation&list_uids=18632557

Choi, S. K., Yoon, S. R., Calabrese, P., & Arnheim, N. (2012). Positive selection for new disease mutations in the human germline: evidence from the heritable cancer syndrome multiple endocrine neoplasia type 2B. *PLoS Genet*, *8*(2), e1002420. https://doi.org/10.1371/journal.pgen.1002420

Eboreime, J., Choi, S. K., Yoon, S. R., Arnheim, N., & Calabrese, P. (2016). Estimating Exceptionally Rare Germline and Somatic Mutation Frequencies via Next Generation Sequencing. *PLoS One*, *11*(6), e0158340. https://doi.org/10.1371/journal.pone.0158340

Keilhack, H., David, F. S., McGregor, M., Cantley, L. C., & Neel, B. G. (2005). Diverse biochemical properties of Shp2 mutants. Implications for disease phenotypes. *J Biol Chem*, *280*(35), 30984-30993. https://doi.org/10.1074/jbc.M504699200

Kinde, I., Wu, J., Papadopoulos, N., Kinzler, K. W., & Vogelstein, B. (2011). Detection and quantification of rare mutations with massively parallel sequencing. *Proc Natl Acad Sci U S A*, *108*(23), 9530-9535. https://doi.org/10.1073/pnas.1105422108

Maheshwari, M., Belmont, J., Fernbach, S., Ho, T., Molinari, L., Yakub, I., . . . Gibbs, R. (2002). PTPN11 mutations in Noonan syndrome type I: detection of recurrent mutations in exons 3 and 13. *Hum Mutat*, *20*(4), 298-304. https://doi.org/10.1002/humu.10129

Martin, J. A., Hamilton, B. E., Osterman, M. J. K., SallyC.Curtin, S. C., & Mathews, T. J. (2015). *Births: Final Data for 2013*

Qin, J., Calabrese, P., Tiemann-Boege, I., Shinde, D. N., Yoon, S. R., Gelfand, D., . . . Arnheim, N. (2007). The molecular anatomy of spontaneous germline mutations in human testes. *PLoS Biol*, *5*(9), e224. https://doi.org/10.1371/journal.pbio.0050224

Rahbari, R., Wuster, A., Lindsay, S. J., Hardwick, R. J., Alexandrov, L. B., Turki, S. A., . . . Hurles, M. E. (2016). Timing, rates and spectra of human germline mutation. *Nat Genet*, *48*(2), 126-133. https://doi.org/10.1038/ng.3469

Segurel, L., Wyman, M. J., & Przeworski, M. (2014). Determinants of mutation rate variation in the human germline. *Annu Rev Genomics Hum Genet*, *15*, 47-70. https://doi.org/10.1146/annurev-genom-031714-125740

Tartaglia, M., Cordeddu, V., Chang, H., Shaw, A., Kalidas, K., Crosby, A., . . . Gelb, B. D. (2004). Paternal germline origin and sex-ratio distortion in transmission of PTPN11 mutations in Noonan syndrome. *Am J Hum Genet*, *75*(3), 492-497. https://doi.org/10.1086/423493

Tartaglia, M., & Gelb, B. D. (2005). Noonan syndrome and related disorders: genetics and pathogenesis. *Annu Rev Genomics Hum Genet*, *6*, 45-68. https://doi.org/10.1146/annurev.genom.6.080604.162305

Tartaglia, M., Kalidas, K., Shaw, A., Song, X., Musat, D. L., van der Burgt, I., . . . Gelb, B. D. (2002). PTPN11 mutations in Noonan syndrome: molecular spectrum, genotype-phenotype correlation, and phenotypic heterogeneity. *Am J Hum Genet*, *70*(6), 1555-1563. https://doi.org/10.1086/340847

Tartaglia, M., Martinelli, S., Stella, L., Bocchinfuso, G., Flex, E., Cordeddu, V., . . . Gelb, B. D. (2006). Diversity and functional consequences of germline and somatic PTPN11 mutations in human disease. *Am J Hum Genet*, *78*(2), 279-290. https://doi.org/10.1086/499925

Tartaglia, M., Mehler, E. L., Goldberg, R., Zampino, G., Brunner, H. G., Kremer, H., . . . Gelb, B. D. (2001). Mutations in PTPN11, encoding the protein tyrosine phosphatase SHP-2, cause Noonan syndrome. *Nat Genet*, *29*(4), 465-468. https://doi.org/10.1038/ng772

Tartaglia, M., Zampino, G., & Gelb, B. D. (2010). Noonan syndrome: clinical aspects and molecular pathogenesis. *Mol Syndromol*, *1*(1), 2-26. https://doi.org/10.1159/000276766

Yoon, S. R., Choi, S. K., Eboreime, J., Gelb, B. D., Calabrese, P., & Arnheim, N. (2013). Age-dependent germline mosaicism of the most common noonan syndrome mutation shows the signature of germline selection. *Am J Hum Genet*, *92*(6), 917-926. https://doi.org/10.1016/j.ajhg.2013.05.001

Zenker, M. (2017). *NSEuroNet* www.nseuronet.com
